# Socioeconomic inequalities in access to maternal healthcare in South-Asian countries: A systematic review

**DOI:** 10.1371/journal.pone.0326130

**Published:** 2025-06-17

**Authors:** Ishrat Binte Aftab, Tisha Chakma, Akash Ahmed, Syed Mohammad Raysul Haque

**Affiliations:** 1 BRAC James P Grant School of Public Health, BRAC University, Dhaka, Bangladesh; 2 Department of Mathematics and Natural Sciences, BRAC University, Dhaka, Bangladesh; 3 Department of Public Health, Independent University Bangladesh, Dhaka, Bangladesh; University of New South Wales, AUSTRALIA

## Abstract

**Background:**

Despite numerous efforts, South Asia is the second most leading region for maternal mortality and morbidity. Although there have been notable advancements on this continent, progress in maternal healthcare access has been slow. Inequalities remain in different maternal health services across diverse population groups. The aim of this study is to better understand the socioeconomic inequalities in maternal healthcare access in South Asian countries.

**Methods:**

Two databases (MEDLINE and Google Scholar) were searched for identifying studies. Studies with data on socioeconomic factors, maternal healthcare, inequality, and South-Asian countries were included. Mixed method appraisal tool was used for quality assessment of the identified studies.

**Results:**

A total of 55 studies from India, Nepal, Bangladesh, Afghanistan and Pakistan were included in the final analysis. Overall, quantitative study showed that maternal healthcare access is linked to economic status, education, occupation, and women’s autonomy. Qualitative studies demonstrated poverty, lack of awareness of complication and service and inability in decision making on healthcare was the reason for not accessing maternal health care. Significant finding from this review includes effect of education mostly evident after secondary education and paternal occupation was significant rather than maternal occupation.

**Conclusion:**

Findings of this review indicates a large disparity in access to maternal health care services. Policymakers should create interventions and financial schemes that are tailored to the mothers from low socioeconomic background.

## Introduction

Globally, maternal deaths are influenced by MHC (Maternal healthcare) access. Maternal death refers to a woman’s death while pregnant, or within 42 days of delivery due to any cause related to pregnancy or its management, excluding unintentional or accidental causes [1]. Approximately 295,000 women died during and following pregnancy in 2017 [2], where Sub-Saharan Africa and South-Asia both were accounted for 86% of these deaths [3]. Alarmingly, everyday 810 women die from preventable pregnancy-related causes [2]. Majority of this causes include, severe bleeding/hemorrhage (27%), infection/sepsis (11%), high blood pressure (14%), complications from delivery, unsafe abortion (8%), embolism (3%) etc. [2,4]. Even though maternal mortality ratio (MMR) declined by 38% (342 per 100,000–211 per 100,000 live births) worldwide, it is yet unsatisfactory [4]. The WHO defines maternal healthcare as health during pregnancy, childbirth, and postpartum along with key markers like, ANC (Antenatal Care), SBA (Skilled Birth Attendance), ID (Institutional Delivery), PNC (Postnatal Care), and EmOC (Emergency Obstetric Care).

South-Asian region, which consists of eight countries including India, Nepal, Bangladesh, Pakistan, Afghanistan, Sri Lanka, Bhutan and Maldives [5]. This region saw a total decline in MMR is from 408 to 134 per 100,000 live births (67%) [6]. However, it still accounts for 47,000 deaths annually,16% of the global total [6]. India has the highest estimated numbers (35,000) of maternal deaths that suffice 12% of global maternal death [7], while while Afghanistan has a very high MMR of 620 in 2020 making it one of the “very high alert” or “high alert” states according to the Fragile States Index [8].

Majority of maternal deaths occur in low- and middle-income countries (94%) where rate for least developed countries were 40 times higher than Europe and 60 times higher than Europe and 60 times higher than New Zealand and Australia [7,9]. Furthermore, the probability of maternal death is 1 in 5300 in high income countries whereas 1 in 49 in low-income countries [3]. In South Asia, except Maldives, all the countries in South-Asia are low or lower middle-income countries [10].

In 2000, the Millennium development goal (MDG) aimed for 75% reduction in global maternal mortality (goal 5a) and universal access to reproductive health (goal 5b) [11]. However, none of the South-Asian countries fulfilled both components of MDGs [12]. Later, Sustainable development goals (SDGs) adopted this to reduce global maternal mortality ratio to less than 70 per 100,000 live births (goal 3.1) and ensuring universal access to sexual and reproductive health-care services (goal 3.7) [13]. To meet the Sustainable Development Goal (SDG) for maternal mortality, annual rate should be 6.4% globally which is 2.1% at present [14]. Except Maldives, none of the South-Asian country is on track to achieve the goals [13]. Acheivingrequires a universal access to good quality maternal healthcare services such as, family planning, better access to high-quality antenatal, intrapartum, and postnatal care by skilled health professionals [9,15,16]. Understanding inequalities in MHC requires distinguishing between access and utilization, as well as inequality and inequity. While access depends on service availability, utilization depends on socioeconomic and systemic factors. Inequality in MHC use becomes inequity when driven by unjust barriers. In South-Asian region, socioeconomic factors related with inequality in access to different components of MHC are household wealth, costs, education, decision-making power etc. [17–22]. Economic inequality is well pronounced in ANC, SBA and EmOC [17,23] with wealthier populations having significantly higher access to ANC 4 + , SBA and ID services in India, Bangladesh, Pakistan, Nepal and Afghanistan [24]. However, in Pakistan women’s autonomy was negatively associated with maternal healthcare [21]. The most pronounced economic inequality persists in SBA and ANC 4 + visits, with double uptake of ANC 4+ in women with education up to secondary or higher than in women with no education [25]. Women autonomy, influenced by socioeconomic status, in healthcare decision making [26]. The patriarchal structure of South Asian household gives limited autonomy to women at household and societal level, resulting in no access to basic health right without permission for husband/senior member [27–30]. Less education and poor self-reported health status were shown to be associated, and health literacy was found to somewhat mitigate this relationship [31]. Mothers often overlook the magnitude of complications due to lack of awareness and end up not accessing MHC services. Cultural beliefs and lack of education in rural areas further limit MHC access, as seen in Nepal where some women use animal sheds for childbirth thinking it is a kind of pollution that might make the household deity angry [32].

While many studies have focused on inequalities in Sub-Saharan Africa, fewer have systematically explored socioeconomic inequalities in MHC access in South Asia. This review aims to investigate these inequalities in South Asian countries.

While many have focused in Sub-Saharan countries [33,34], other studies explored specific MHC indicator, country [35–37], factors etc. [17] However, a systematic regional analysis of socio-economic inequalities affecting access to maternal healthcare across all South Asian countries are absent. In light of this, this study aims to systematically review and synthesize existing literature to provide a better understanding of the factors associated to the inequalities in access to maternal healthcare in South Asian countries. By doing so, it contributes to the evidence base needed for policy interventions that can address disparities and improve maternal health outcomes in this region.

### Access vs Utilization and Inequality vs Inequity

“Access” refers to an individual’s ability to receive healthcare [38], while “utilization” presumes that access exists and receipt of service or recommendations from a healthcare plan [38]. Access determines people’s potential to use the health service which is directly connected to their utilization power [39]. Then again, people do not utilize the healthcare due to access problem in healthcare [40]. As a result, “utilization” of health care is frequently used as an operational proxy for “access” to health care in developing countries and this review includes both terms for identifying papers [41].

Inequality refers to observable inequalities in health between subsets of a population, while inequity is a normative term that refers to the avoidable and/or unfair differences in health among demographic segments. This review includes both terms, as observed inequalities often reflect inequities in healthcare access [25].

### Dimensions of “access to healthcare”

There is no universal definition of access [42]. Penchansky and Thomas (1981) demonstrated five dimensions of health care access: accessibility, affordability, accommodation, availability, and acceptability [43]. **Accessibility** refers to geographic accessibility, **availability** assesses whether the provider has the necessary resources, **affordability refers to the cost of the service**, **accommodation** involves meeting client preferences and **acceptability reflects** relationship between clients’ expectation and provider characteristics [43]. This review focuses on four dimensions—availability, geographic accessibility, affordability, and acceptability— as adapted by different literature, to examine inequalities in maternal healthcare access [40].

## Methods

The protocol for this systematic review was registered on PROSPERO, in which the inclusion criteria and methods of analysis were specified: registration number:CRD42022359879. The primary search was done in MEDLINE through PubMed as it includes comprehensive index of biomedical literature, ensuring high-quality peer-reviewed studies. Google scholar was searched manually to add additional relevant literature not indexed in MEDLINE. Relevant studies from reference were also searched.

### Eligibility criteria

Primarily socioeconomic factors and maternal healthcare indicators were selected for inclusion criteria. Socioeconomic factors were economy (economic position or income of the mother, father, or family), education (maternal and paternal), wealth (individual or household status) and occupation (maternal and paternal). Maternal healthcare indicators were ANC, PNC, SBA, ID and EmOC. Studies that explored access dimensions (geographic accessibility. affordability, availability, and acceptability) were also included. Studies conducted in South-Asian countries between 2000 and 2024 that were published in English, were considered for inclusion. This time frame was selected as most all the development goals were set from 2000 and condition of maternal healthcare after taking such initiative is important. Quantitative studies were mainly the cross-sectional analytical ones, were included to comprehend the magnitude of the problem; while qualitative and mixed method studies were added to understand users’ and service providers’ views on the potential reasons for inequality. Other types of studies including scoping, systematic or narrative reviews were avoided to keep the findings simple and straightforward. Studies on abortion, contraception, and cesarean sections were excluded due to their complex sociocultural contexts, which fall outside the focus of this review on socioeconomic factors in MHC access [44,45]. Similarly, studies on indigenous, tribal, and migratory populations were not included, as their healthcare inequalities are often influenced more by geographic and cultural factors than purely socioeconomic ones [46–48].

### Search strategy

The database MEDLINE was searched for five different key terms, such as socioeconomic factors (economy, education, occupation, wealth), maternal healthcare (ANC, SBA, PNC, EmOC), inequality (inequity, inequality, disparity, barrier), access dimensions (accessibility, affordability, availability, acceptability) and country (South-Asian region). MeSH terms, synonyms for the main concepts were used as keywords and subject headings in the selected databases. The detailed search code for MEDLINE is included in the S1 File. Google scholar was searched manually with key terms such as, “socioeconomic inequality”, “maternal healthcare”, “access”, “utilization”, “South-Asian countries”. First 10 pages of googles scholar was taken into account since after that relevant results were not found.

### Data extraction process

Database search from MEDLINE was extracted 15^th^ March 2024 and from google scholar on 30^th^ March 2024. All studies were initially exported in Rayyan software. After merging duplicates were removed. Initial elimination was done based on title only. After primary screening was done, abstracts were checked for inclusion criteria. Further in-depth screening was then done to identify articles meeting eligibility criteria by reviewing the methodology sections for each publication. Two reviewers searched independently and the eligible studies were cross checked by both. Following this screening process, the Preferred Reporting Items for Systematic Reviews and Meta-Analyses (PRISMA) flowchart (S3 File) was adapted to assort resulted articles into identified papers, screened publications, and included and excluded articles with specific reasons [49]. The principal outcome was any socioeconomic inequalities that are faced in access to maternal healthcare in this region. Data regarding socioeconomic inequalities such as “Education”, “Occupation”, “Wealth”, “Women autonomy” were sought regarding maternal healthcare. Any disagreement during the study selection and data extraction process were resolved through discussion.

### Study assessment

There were three different study types (cross sectional, qualitative, mixed method) included in the review. There are numerous validated quality assessment tools to assess cross sectional and qualitative studies but none for mixed method study. Only Mixed Method Appraisal tool (MMAT) allowed to assess all three types of study. Thus, to keep the consistency in the study assessment criteria Mixed Method Appraisal tool (MMAT) was used. MMAT is a reliable assessment tool for mixed method studies in healthcare research, particularly for systematic reviews [50]. For each study design it has two basic screening question in general and five specific questions for appraisal. Papers were sorted into “Good” (100%), “Moderate” (80%) and “Poor” (≤60%) categories for better understanding. The assessment tool was only used for the appraisal of the studies. Thus, all the papers were included for result synthesis, regardless of their assessment score. Quality appraisals were conducted by two reviewers independently. A detailed result provided in the S2 File.

### Data synthesis

The quantitative results were described by socioeconomic factors. Table 3 has information with details on the study, sample size, indicator of MHC, significant result and MMAT score. To synthesize qualitative results, thematic analysis was used to uncover the underlying reasons for lack of MHC access. There are different versions of thematic analysis, and it has a broader degree of theoretical flexibility, that makes it easy to analyze [51]. Small summary of each finding is also added to provide an overall view in the supplementary material.

## Result

**Fig 1:**
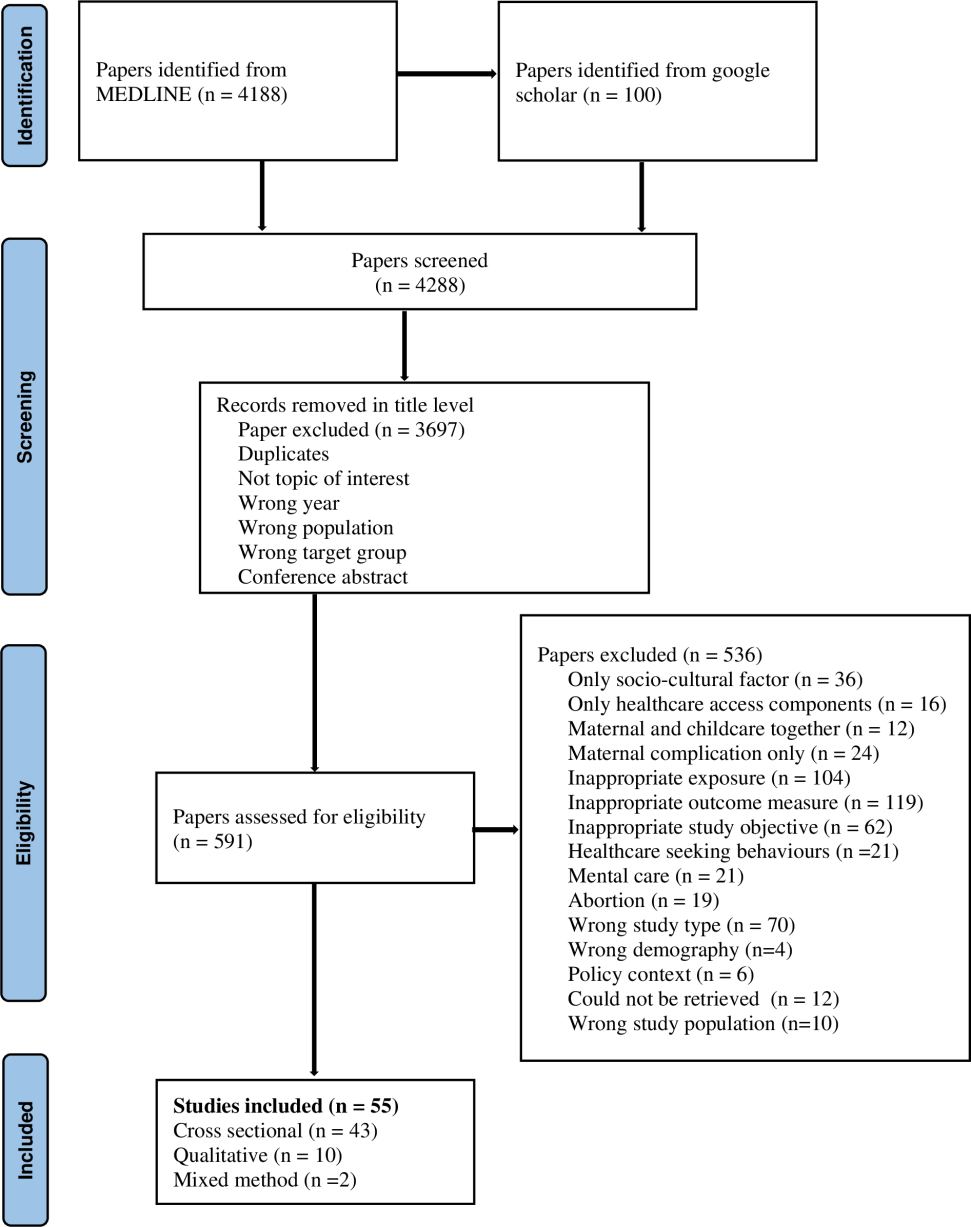
PRISMA flowchart for identification of studies.

The systematic literature search resulted in a total of 4288 records. After deletion of duplication in both database and based on titles, 591 studies remained (excluded, n = 3697). From reading the abstract/full article based on pre-defined criteria, 536 papers were excluded and 55 papers were eligible for the systematic review, as illustrated in Fig 1.

### Characteristics of the included studies

Of the 55 articles, 43 (78.18%) were cross sectional analytical, 10 (18.18%) were qualitative and 2 (3.64%) were mixed-method studies. Studies from five countries of South-Asia was found to be eligible to include in the review. Additionally, three cross-sectional papers that presented findings across multiple countries were included. Instead of counting papers for each country, the following table counts papers once for consistency. However, the results are displayed individually for each country in the result table. Among the 43 quantitative studies, 35 studies reported economy [37,52–85], 33 studies reported education [52,54,56,58–61,63–71,73–75,77–90], 14 studies reported occupation [52,54,60,61,64,66,70,71,73,74,77,82,87,91] and 11 studies reported about women autonomy [52,64,66,71,73,74,77,83,88,92,93]. Among the 10 qualitative papers, 10 studies reported economy [94–103], while 4 studies focused on education [95–97,100] and 4 studies examined women autonomy [94,95,100,103]. Both of the mixed method studies reported economy [104,105]. One study described three South-Asian countries (Nepal, Bangladesh and Pakistan) and is counted once in the Table 1 to keep consistency for study number [73]. Additionally, two studies [82,83] examined Bangladesh and Pakistan; these studies are counted as two instead of four in order to maintain consistency for study number in Table 1.

**Table 1:** Number of total studies and study types included in the review by country.

Country	Total study	Cross sectional	Qualitative	Mixed method
India	12	10 [37,52–57,80,85,92]	2 [94,95]	–
Bangladesh	17	15 [62–68,73,74,76,77,82–84,90]	2 [98,99]	–
Nepal	11	9 [58–61,81,86,87,91,93]	2 [96,97]	–
Pakistan	8	4 [69,70,75,78]	3 [100–102]	1 [104]
Afghanistan	7	5 [71,72,79,88,89]	1 [103]	1 [105]

The Majority of the quantitative studies conducted secondary analysis using demographic and health survey data, resulting in large sample size. For understanding the impact of socioeconomic position, most of the studies measured and used wealth index/quintile and rich-poor ratio/gap. Very few compared with household income as demographic health surveys do not collect data on this to measure socio-economic position. Most qualitative studies were regional exploration of inequalities, especially in rural counterparts of the country.

### Summary of the findings

Household wealth status and position in wealth quintile was strongly associated accessing MHC services in India, Nepal, Bangladesh, Pakistan, and Afghanistan. A significant rich-poor gap ratio was also evident in the service utilization. Education had a positive impact on accessing MHC, however, many studies demonstrated this impact was more significant after secondary or higher education. Health literacy played a crucial role in accessing different care components. Due to lack of awareness of the service and complications of pregnancy, women did not utilize MHC, resulting in some very unhealthy and dangerous medium of delivery practice. Only a few studies found women’s employment status significant for MHC utilization, whereas husband’s occupation was more influential factor. Women autonomy played a vital role in access part in these countries of South-Asia, especially decision-making autonomy on healthcare. In many cases, women were restricted by the decision of husband, elderly, or mother-in-law for utilizing MHC.

The findings of the qualitative studies were categorized into three key themes related to socioeconomic factors that acted as barriers to MHC access. They were organized under the theme of “Economy” if the study described financial constraint, poverty etc.; “Education” if the study highlighted on health literacy, lack of awareness of complication, lack of awareness of service etc.; “Women’s autonomy” if the study talked about lack of decision-making power on healthcare utilization for not accessing MHC.

### Quantitative findings

Quantitative findings are explained briefly in Table 2 while detailed results are available in the S4 File.

**Table 2:** Quantitative findings summary.

Sl No	Author_ Year of publication	Sample	Factors	Key findings	MMAT
Study type: Cross sectional					
1	Singh A, 2012 [37]	216831	Economy	Rich-poor ratio positively significant with utilization of ANC and PNC.	100%
2	Pallikadavath S, 2004 [52]	11369	Economy, Education, Occupation, Autonomy	Standard of living positively significant with home visit, not very significant without home visit. Mostly with at least secondary education have significance with ANC utilization. Working status: slightly positive significant for Uttar Pradesh for both including and excluding home visit. High autonomy has slightly positive significance only in Madhya and Uttar Pradesh, excluding home visits.	100%
3	Prakash R, 2013 [53]	82837	Economy	Economic status positively significant with the utilization of ANC and SD.	100%
4	Sridharan, S, 2017 [54]	5666	Economy, Education, Occupation	Wealth index, working status and husband’s education strongly significant with ANC utilization.	100%
5	Krishnamoorthy Y, 2020 [55]	190797	Economy	Overall not much significant; high degree of inequality for ANC 4+ in Central and Eastern region.	100%
6	Zuhair M, 2017 [56]	36447	Economy, Education	Wealth quantile and education are positively significant with utilization of ANC.	100%
7	Awasthi A, 2016 [57]	3104	Economy	High coverage gap in lowest wealth quintile in utilizing ANC and SBA.	100%
8	Chalise B, 2019 [58]	2086	Economy, Education	Economic position and education are positively significant with with ANC, SBA and PNC use.	100%
9	Dhakal S, 2011 [59]	150	Economy, Education	Not utilizing ID due to lack of money and literacy in Nepal.	100%
10	Khanal V, 2014 [60]	4079	Economy, Education, Occupation	Positive significance of economic status and education with the use of PNC. Women working in agriculture less likely to use PNC while paternal occupation (professional/ manual) positively associated with PNC use.	100%
11	Joshi C, 2014 [61]	4079	Economy, Education, Occupation	Wealth index and Husband’s occupation positively significance with ANC use. Both maternal and husband’s education are significant with ANC use.	100%
12	Pulok MH, 2018 [62]	4483	Economy	Positive significant; Pro rich inequality in all regions; especially Barishal, Chittagong and Sylhet of Bangladesh.	100%
13	Huda TM, 2019 [63]	28032	Economy, Education	Wealth index and maternal education positive significant with institutional delivery in Bangladesh.	100%
14	Dalal K, 2012 [64]	4925	Economy, Education, Occupation, Autonomy	Home delivery increases with lower SES and lower level of maternal and paternal education. But, women’s working status has no association with the increase of home delivery. Women’s autonomy in spending is not significant.	100%
15	Kamal SM, 2015 [65]	4809	Economy, Education	Richest economic status and women’s education (at least secondary) are highly significant with the utilization of SBA.	100%
16	Pulok MH, 2016 [66]	3730 (2004) 3365 (2007) 4648 (2011)	Economy, Education, Occupation, Autonomy	Positive significant; especially richer and richest group; mother’s and husband’s education; microfinance program positively significant only for ANC. Besides, Employment status: not positively significant with ANC and ID; slightly positive significant with SBA. Besides, Healthcare decision making: positive significant only for ANC 4 + .	100%
17	Chanda SK, 2020 [67]	4475	Economy, Education	Wealth index and education; positive significant. Richest have higher tendency to complete ANC 8 + visit; maternal education for ANC contact and ANC 8 + ; paternal education was only for ANC contact.	100%
18	Yaya S, 2017 [68]	7313	Economy, Education	SES, maternal and paternal education; positive significant with ID.	100%
19	Jain AK, 2015 [69]	4435	Economy, Education	Positive significant; specially for upper middle and highest household wealth status and education above secondary level.	100%
20	Sahito A, 2018 [70]	7142	Economy, Education, Occupation	Wealth index; positive significant with ANC utilization. Also, positive significant; for maternal education in GB, Sindh and Punjab; for paternal education only in KPK and Husband’s occupation: positive significant; only in Sindh Province.	100%
21	Mumtaz S, 2019 [71]	19642	Economy, Education, Occupation, Autonomy	Socio-economic status, education and women’s working status positive significant towards ANC and SBA use in Afghanistan. Decision making autonomy on husband’s earning has positive significance.	100%
22	Akseer N, 2016 [72]	21290	Economy	Inequality exists in lower SES.	100%
23	Rahman MA 2021 [73]	16429; 3962 (Nepal) 4278 (Bangladesh) 8189 (Pakistan)	Economy, Education, Occupation, Autonomy	Household wealth quantile highly associated with facility-based delivery. Secondary and higher education of women positive significant both for Nepal, Bangladesh and Pakistan; husband’s education not significant in Nepal and Pakistan. Only higher education of husband positive significant in Bangladesh. Professional or service job of husbands positive significant in Nepal. Where, both Women’s and 24husband’s occupation have moderate significance in Bangladesh. Decision making power in women’s health is not much significant in any of three countries.	100%
24	Methun MIH 2022 [74]	4012	Economy, Education, Occupation, Autonomy	Maternal secondary and higher educational status and Socio-economic status positively associated with MHC usage while Women’s working status not significant. Strong relationship between women’s decision-making autonomy and the use of ANC care during pregnancy and PNC; however joint decision making with partner is significant.	100%
25	Budhwani H, 2015 [75]	7399	Economy, Education	Household wealth status is Positive significant; specially for ANC 4 + , ID and SBA. Education has positive association with ANC/ Prenatal, SBA, ID and PNC service utilization in Pakistan.	100%
26	Zere E, 2013 [76]	11178	Economy	Wealth related inequalities favoring wealthiest segment.	80%
27	Bhowmik J, 2019 [77]	17863	Economy, Education, Occupation, Autonomy	Wealthier respondents and parents with higher education and working status have higher tendency to use ANC and SBA. Women’s participation in household decision has positive significance.	80%
28	Ghaffar A, 2015 [78]	2339	Economy, Education	Wealth index and literacy estimate has positive significance with ANC use.	80%
29	Azimi MW, 2019 [79]	18790	Economy, Education	Wealth status and both maternal and paternal education are positively significant with ANC use in Afghanistan.	80%
30	Thakkar N, 2023 [80]	172702	Economy, Education	Women with no formal education and those from the poorest households were at highest risk of inadequate ANC.	100%
31	Thapa B, 2023 [81]	1932	Economy, Education	Women from poorer household are less likely to deliver in a health institution. Women’s education is a significant predictor of ID.	100%
32	Misu F, 2023 [82]	Bangladesh (4948) Pakistan (5122)	Economy, Education, Occupation	In Bangladesh, there was greater inequality in skilled birth attendance (SBA) than other maternal healthcare services based on wealth, women’s education, husband’s education. In Pakistan, inequality was highest in at least four ANC by skilled providers by wealth, women’s education, and their husband’s education. Women’s employment-related inequality was highest for ID in Bangladesh, while in Pakistan, it was highest for at least four ANC visits by the skilled provider.	100%
33	Misu F, 2023 [83]	Bangladesh (4440) Pakistan (3780)	Economy, Education, Autonomy	In Bangladesh and Pakistan, inequality in PNC check of women was highest by wealth, women’s education. In Bangladesh and Pakistan, inequality was present for women’s autonomy favouring women who had the autonomy to make decisions alone or jointly with their husbands in receiving PNC service.	100%
34	Methun MIH, 2023 [84]	5012	Economy, Education	Household wealth status and maternal education contributed the highest to inequality in accessing adequate maternal healthcare services such as receiving at least four antenatal care (ANC) visits, and ID.	100%
35	Singh L, 2019 [85]	190898	Economy, Education	Wealth index and education have positive significance with ANC utilization in India.	100%
36	Bhatta DN, 2015 [86]	2178	Education	Paternal education and wealth status is positive significant; inequality persists in lower SES.	100%
37	Khatiwada J, 2020 [87]	4400	Education, Occupation	SBA utilization has positive significance with education and occupation other than agriculture.	100%
38	Yeo S, 2022 [88]	11056	Education, Autonomy	Education significant for ANC service utilization in Afghanistan. Also, Women taking health care decisions alone or jointly has positive impact in ANC Utilization.	100%
39	Stanikzai M.H., 2023 [89]	6227	Education	The likelihood of receiving 5–8 ANC services was significantly greater for pregnant women who could read and write, and women whose husbands could read and write.	100%
40	Jannat Z., 2023 [90]	ANC= 245 PNC = 133	Education	Women with higher education were more likely to utilize maternal health care service(ANC, PNC). Monthly expenditure did not exert any statistically significant impact on receiving maternal healthcare services.	100%
41	Dhakal S, 2007 [91]	150	Occupation	Women with any job other than farming and husband having formal jobs mostly use PNC	60%
42	Mistry R, 2009 [92]	11648	Autonomy	Financial autonomy has positive significance; mainly significant in South region; not very much in North and East of India.	100%
43	Kc S, 2016 [93]	4148	Autonomy	Women who have autonomy over spending husband’s money prefers institutional delivery.	100%
Study type: Mixed Method					
44	Ansari MS, 2015 [104]	690 (Household survey); 309 (Exit survey	Economy	High travel cost even to the public facility.	80%
45	Higgins-Steele A 2018 [105]	2479	Economy	Money for health service was the top reason to no attendance.	60%

### Economy

Economic disparity was highly pronounced in India, both nationally and regionally. Economic position—measured through wealth quintile, wealth index, household income, or rich-poor ratio—was positively associated with receiving adequate ANC [54–57,80,85] and PNC [37]. However, some exceptions were also seen, i.e., inequality was less for iron and folic acid supplement [55,56]. Only a few studies have (n = 2) have assessed the economic inequality in state level. Gap of utilization for ANC 4 + was higher in central, eastern states [55]; for ANC and SD in Uttaranchal, Uttar Pradesh, Bihar, Jharkhand, and Madhya Pradesh [53].

Similar to India, inequality in utilizing ANC, SBA, PNC, and ID was explored and found significantly associated with socioeconomic position in Bangladesh [62,64–68,73,74,76,77,82–84], Pakistan [69,70,73,75,78,82,83], Nepal [58,59,61,73,81,86] and Afghanistan [71,79,106]. In contrast to that, household monthly expenditure was not an independent factor for utilization of ANC and PNC among rural pregnant woman where as women’s association with microfinance program was insignificant in uptake of delivery care in Bangladesh [64,90]. Unlike other countries, a mixed method study reported travel cost to EmONC facility was unaffordable for both public and private hospitals in Pakistan [104].

### Education

Effect of maternal and paternal education was found significant in accessing MHC services especially ANC and ID in most of the South-Asian countries. Maternal education positively affected the utilization of ANC [52,54,56,80,85] in India; ANC [58,61,93], PNC [60], SBA and ID [58,59,73,81,86,87] in Nepal; ID [63,64,66,68,84], SBA [65,66,77], Place of delivery [73], ANC [66,67,77,84], PNC [83] in Bangladesh; and ANC and SBA [71,79,88] in Afghanistan. Likewise, in Bangladesh, higher levels of maternal education were linked to better utilization of MHC services [74,84]. Almost all four components of MHC (ANC, SBA, ID and PNC) were positively associated with higher maternal education level, awareness and knowledge of available MCH services in Pakistan [73,75]. Furthermore, inequity was largest in at least four ANC by skilled providers by the education of the women and their husbands in Pakistan, across various maternal healthcare services [82]. Notable association of MHC services utilization apparently after secondary education of mother was reported in the South-Asian countries [52,59,63,69,85]. Similar to the findings of maternal education, inequality persists based on the literacy rate and education level of husbands in access to the MHC services, i.e., ANC in India [54]; ID and PNC in Nepal [60,86]; ANC in Afghanistan [79,89] and ANC, SBA, ID and PNC in Bangladesh [64,66–68,73,77] etc. Besides, maternal education was significant for ANC utilization in all provinces of Pakistan except in Baluchistan and Khyber Pakhtunkhwa [70].

### Occupation

Women’s employment status was found to be a significant factor in accessing MHC services, particularly in India, where it was notably significant in Uttar Pradesh [52,54]. In Nepal, women’s occupation, other than agriculture had higher odds of using SBA [87] and PNC [91] and husbands doing formal sector job/working abroad or professional/ manual occupation were more likely to attend PNC [60,91] and less likely to prefer home delivery [73]. In contrast to them, SBA and place of delivery had positive association with occupation in Bangladesh [73,74,77]. However, employment-related inequalities varied by country. In Bangladesh, employment inequality was most pronounced for ID, whereas in Pakistan, it was highest for accessing at least four ANC visits by a qualified provider [82]. Additionally, compared to women who are not employed, working mothers had greater likelihood of receiving appropriate ANC contact in Bangladesh [64,66]For Pakistan, husband’s occupation was significant factor in uptake of ANC; only in Sindh province [70]. On the other hand, women’s employment status was not associated with accessing ID in Pakistan [69,73]. However, ANC and SBA utilization was positively associated with women’s working status in Afghanistan [71].

### Women’s autonomy

Beyond economic position, education and occupation, women’s autonomy in making decisions on household expenses and utilizing MHC also contributed to inequalities in access to MHC in the South-Asian countries. Regional variation in women’s autonomy and inequality to access in MHC services were observed in India and Nepal. In India, women’s financial autonomy was associated with “delivery by a trained person” (South), “postnatal checkup” (South), “institutional delivery” (North, east, south) [92]. However, Pallikadavath (2016) found significant association with the odds of ‘high autonomy’ of women in receiving ANC in Uttar Pradesh and Madhya Pradesh [52]. In Nepal, ANC and SBA access increased with high autonomy in large household purchases and decisions regarding money earned by their husbands [93]. Similar like Nepal, women autonomy was related with ANC and SBA uptake in Bangladesh. However, the type of autonomy was different. Significant association was reported for women’s autonomy on healthcare decision making with ANC [66,77] and SBA [77]. Furthermore, inequality exists regarding women’s autonomy in Bangladesh and Pakistan, favoring those who were able to make decisions about obtaining PNC checks for women either on their own or in jointly with their husbands [83]. On the other hand, no observable association between ID and women’s decision-making autonomy on spending money was reported [64]. However, Rahman and his colleagues hadn‘t found any significant association of decision making autonomy in three South-Asian countries [73]. Conversely, in Afghanistan, women’s decision-making autonomy on spending husband’s earnings was associated with ANC 4+ and SBA utilization [71].

### Qualitative findings

As mentioned in the literature, several indirect factors stem from socioeconomic factors. For example, health literacy, lack of awareness of complication or service was not directly socioeconomic factors as such. However, they are the result of illiteracy or lack of education. Three themes emerged from the qualitative findings that is acting as a barrier for accessing MHC in these five countries. The summary of the findings is described in Table 3.

**Table 3:** Qualitative findings summary.

Sl No	Author_Year of publication	Sample	Themes	Key findings	MMAT
1	Vidler M, 2016 [94]	335	Economy, Women’s autonomy	Financial constrains remains barrier for routine check ups. Mother in-law and husband takes decisions for healthcare services.	100%
2	Mahapatro M, 2015 [95]	120	Economy, Education, Women’s autonomy	Transportation and financial constrains is a crucial determinants to avail MHC. Unequal treatment and biasedness of health workers regarding economic position and literacy. Women mostly doesn’t have the decision making autonomy regarding MHC utilization.	100%
3	Milne L, 2015 [96]	20	Economy, Education	Improper utilization of birthing facility or delivery care due to financial obstacles, lack of education and awareness regarding MHC services.	100%
4	Lama S, 2014 [97]	6 FGD (8–10 people), 10 IDI	Economy, Education	Lack of transportation facilities, literacy, awareness and low socioeconomic status are the barriers of proper MHC utilization.	60%
5	Afsana K, 2004 [98]	170	Economy	Unofficial costs and corruption to avail proper obstetric care in hospitals.	60%
6	Pitchforth E, 2006 [99]	39	Economy	Unwanted informal service costs and financial constrains in utilizing EmOC.	100%
7	Nisar YB 2016 [100]	83	Economy, Education, Women’s autonomy	Financial barrier, travel cost, expensive service charge for private services and lack of education are reasons for not accessing ANC. Mother in-law and husband take decisions for ANC check ups.	100%
8	Mumtaz Z, 2014 [101]	163	Economy	Poor economic status is a barrier to avail SBA and ID. Unofficial cost, travel costs and lack of awareness regarding ANC, ID and PNC are also obstructs MHC utilization.	100%
9	Memon Z, 2015 [102]	60 FGD	Economy	Transportation and medicine costs hinders the utilization of ANC, PNC, ID.	100%
10	Rahmani Z, 2013 [103]	27	Economy, Women’s autonomy	Poor economic condition is impediment of MHC utilization. Husband and In laws are the decision maker in the house and give permission for ANC visit mostly in critical condition.	100%
Study type: Mixed Method					
11	Ansari MS, 2015 [104]	3 FGD (6–8 people in each)	Economy	Poor economic condition, expensive emergency services and transportation causes less utilization of MHC services.	80%
12	Higgins-Steele A, 2018 [105]	9FGD and 16KII	Economy	Financial expenses are the barrier to utilize ID.	60%

### Economy

Across all five countries, financial constraints or poverty were consistently reported as barriers to accessing various components of MHC. Financial barrier was impeding the utilization of MHC in different regions of India [94,95]. That includes transportation cost for routine care because it required hiring private vehicle which was unaffordable [94]. Biased and rude behavior towards rural poor mother was also impeding the utilization of delivery service [95]. In Nepal [96,97] and Afghanistan [103,105] mothers did not access MHC services due to financial costs and constrains. Participants in Bangladesh reported unofficial or hidden costs resulting from corruption was a huge barrier in accessing MHC. This unofficial cost includes tipping the cleaning staff, taking the mother to the operation room or for admission to the clinic [98,99]. Qualitative findings from Pakistan showed financial limitations in utilizing MHC was due to under-the-table payments, limited access to emergency transportation, physical inaccessibility of hospitals, treatment, medicine, and consultation cost (private facility) [100–102,104]. Similar like India, hospital stuffs discriminated based on socioeconomic position of the patients in consultation, treatment and even in emergency conditions [104].

### Education

Several issues stemming from a lack of education—such as poor health literacy, lack of awareness about available services, and unawareness of pregnancy complications—hindered access to MHC. The result was of similar pattern in four countries except Afghanistan did not have any qualitative findings in this regard. Verbal abuse and biased behavior against illiterate women also a reason for not utilizing MHC services in India [95]. Similarly, in Nepal and Pakistan, illiteracy or lack of awareness were perceived as significant barriers to accessing MHC [96,97,100,102].

### Women’s autonomy

Overall, in South-Asian context women’s decision-making autonomy on health was very less as family structures were mostly patriarchal. Four countries of this region, India [94,95], Pakistan [100], and Afghanistan [103] reported that decisions regarding delivery, ANC and other MHC services were taken by elderly, mother-in-law, or husband, therefore, restricting women from utilizing the service.

### Discussion

To the best of the authors’ knowledge, this is the first systematic review to examine socioeconomic inequalities in accessing maternal healthcare (MHC) in the South Asian region. Almost all quantitative findings could be generalized nationally as most of the studies used demographic health survey data and sample size was big. In contrast, while qualitative findings were drawn from studies conducted in specific rural areas, they provide in-depth insights into the lived experiences of women facing barriers to MHC. These qualitative studies, despite smaller sample sizes, offer rich contextual understanding that complements the broader patterns identified in the quantitative analyses.

No relatable studies were found in Maldives, and Sri Lanka. This can be explained by previously mentioned literature. These two countries along with Bhutan have highest coverage for ANC, SBA and ID compared to other five countries [107–109]. Another possible reason behind this is the out-of-pocket-expenditure is not very high in Maldives resulting in insignificant socioeconomic inequalities in MHC access [110]. Besides, both countries had a higher decline in MMR from 1990 to 2015 [111]. Individually, Maldives has achieved remarkable improvement in maternal health in recent years. The MMR decrease from 1990 to 2015 was 9.2 in Maldives whereas the annual decrease globally was only 2.3 [112].

Overall, socioeconomic factors contributing in access to MHC were economic position, education, occupation, and women autonomy. This finding was mostly consistent except occupation from another systematic review on inequalities in MHC utilization developing countries [17]. Majority of quantitative studies explored and reported inequality in ANC and SBA services. Previous studies demonstrated the similar result [110,113].

Most of the studies included in this review have found household wealth status/wealth index/wealth quintile was associated with MHC access. This finding is aligned with wide range of literature from low-income and developing countries [18,19,21,22,114–117]. The qualitative findings also support this result. They reported poverty as the barrier for not utilizing MHC. Another qualitative study conducted in rural areas of Zimbabwe also reported the same [118]. As a reason for not utilizing MHC, they mostly mentioned costs for transportation, medicine service and under the table payments. Few of them also reported that informal payments were taken from them forcefully, otherwise hospital staff refused to provide service. Previous studies indicated transportation, medicine cost as well as informal cost as a barrier for utilizing MHC in low- and middle-income setting [119,120]. Besides, for poor people, rather than accessing MHC it is feasible to buy food and cloths. This makes them to go for cheap services such as traditional birth attendants but not using ID or SBA [77]. Engagement to microfinance (MFI) program is another significant aspect to assess socioeconomic position of mother. According to a report of World Bank, 17% of the households in South-Asian region are facilitated by MFI program [121]. However, only one study from Bangladesh assessed this factor and found a negative association. Literature affirms the fact that coverage of MFI is low in this South-Asian region. In 2005, the total demand for microcredit was $15 billion, but only $2.3 billion was given to the poor [121]. However, the impact of MFI is somewhat inconclusive in South-Asia. Another important finding from qualitative studies was that mothers from low socioeconomic position were treated badly and prioritized less compared to rich people. Literature from low and low-middle countries are in line with this finding [122–124]. This can also be linked with the supply side inequality in access to MHC as stated before in the review.

Maternal education was an important socioeconomic factor contributing in inequality to MHC access. Findings from quantitative studies affirmed that and it is also aligned with literature [18,21,115–117,125]. On the other hand, most of the qualitative findings indicated lack of awareness about service and complication as the barrier for not using MHC. Besides, due to lack of health literacy MHC was not a felt need to them as they considered this as a natural event. Two systematic reviews on barriers in accessing MHC in low-income countries had similar findings [119,126]. Studies demonstrated formal education had the potential to impact the utilization of health-care facilities in a variety of ways [127]. Women with higher education have more access to written material, and they are more knowledgeable about healthcare services [128,129]. Also, an educated woman can better catch health messages delivered through newspapers, billboards, and other media, resulting in higher health education [130]. Then again, formal education challenges traditional beliefs about health and health-seeking and transforms women’s attitudes towards accessing MHC care [130,131]. One important finding from this review is that impact of maternal education on MHC utilization is significant only after secondary education. Even though this finding is similar with the findings from WHO report on state of inequality in developing countries, it does not explain the reason [25]. Possible reason can be that girls do not get reproductive health information in primary education in South-Asia. Also, teachers feel uncomfortable discussing this chapter as this is a taboo, especially in front of male students making it unavailable for young girls [132]. Then again, most of the curriculums include reproductive health knowledge from 9^th^ grade or from 11^th^ grade in some countries. To get all information on reproductive health formally, it takes at least 12 years of education in this region. On the other hand, a sizable portion of girls get married before age of 15, leaving them unaware about the necessary information and complications of pregnancy. The reproductive health education can improve knowledge and reduce reproductive health problems among adolescents in developing countries [127]. Previous studies also reported that educated male had better knowledge about the benefit and awareness of the service utilization [19,133,134]. Part of the reasons behind that is because of the patriarchal structure of the families in South-Asia, husbands decide where to spend money and if they are educated, they access MHC services if needed [135]. Moreover, educated husbands have a good communication with their spouses about availing maternal healthcare in developing countries [128,129].

This review found paternal occupation significantly associated with MHC service utilization, but maternal occupation was not that much. However, few literatures suggest, paternal and maternal occupation has association with MHC utilization [114–116]. Review finding was otherwise might be because most women in low socioeconomic position was housewife or engaged in agriculture. Even if they were working, in most cases they did not have the autonomy on that. Furthermore, the significance of occupation was mostly evident in case of formal jobs or any job other than agriculture. Having agriculture as occupation was either not significant or negatively significant. The underlying reason can be formal job exposes them to outside world and they learn more about maternal health and service information. Literature also suggests, women who work in fields other than agriculture may be able to earn more money in cash, allowing them to make more independent decisions and access maternal services [87]. Conversely, most rural women who primarily work in agriculture do not receive cash to spend on their health [87].

Women autonomy was the least assessed form of socioeconomic inequality overall. Quantitative analysis demonstrated women autonomy especially in household decision making was an important factor in accessing MHC in South-Asia. Previous findings from low-income and developing countries are in line with this finding [92,115,116,136] On the other hand, qualitative findings indicated women’s less autonomy on healthcare decision making was a significant barrier in accessing MHC. Most of the times decisions were taken by husband or mother-in-law restricting them to access health facility services. Literature also suggested as due to the dominant patriarchal mindset and as the sole earning member of the family, husband, and husband’s female family member especially, mother-in-law’s decision was a determining factor in women’s ability to seek for institutional care [137,138]. In Bangladesh, 35.6% of women mentioned in-laws’ objections and 17% mentioned husbands’ objections to obtaining medical help during medical emergencies [137]. This is often the case in South-Asian household. For example, husbands and elder female in-laws in Pakistan frequently defined women’s treatment needs, such as which services were required, which health concerns required medical attention, and whether household money should be spent on health care [137]. One significant factor to consider in this study is that, women autonomy is a complicated term and indicators to calculate this vary widely based on context and country. Even after coming from a wealthy background, women can have little control over their healthcare decision-making. Mostly because mothers-in-law have preconceived view that their daughters-in-law should deliver without medical attention, just as they themselves had done in the past [97]. Husbands have the similar mindset comparing their wives with their mother [97]. In more religious settings, such as Afghanistan, women needed husband’s permission even to go out from home and they were not allowed to be alone in public [71,79]. For this reason, as most of the times male members were busy outside, women could not go for health checkups. This systematic review suggests even though women autonomy was the least studied factor, it is one of the most concerning socioeconomic factors for South-Asia. This region deals with the double burden of barrier – patriarchy and religion which is shaping the pathway of women autonomy.

Sri Lanka’s approach towards maternal health can be a good example for fighting inequality to MHC access for other countries in this region as it shares similar socio-demographic characteristics. Despite having low GDP compared to other countries of South-Asia, they have the highest decline in maternal mortality. Approximately 89% of adult women were literate in Sri Lanka compared with 43% of South-Asian average. This naturally increases access to MHC. On the other hand, its public health infrastructure is very strong focusing on access especially for vulnerable groups; a viable referral system with accessible transport option; quality improvements etc. Moreover, the most distinguished approach they have taken is professionalization of midwifery. Sri Lanka have provided specialized training to midwives to gain clinical competence, are licensed or authorized by government agencies, and are provided with support in the form of supplies and monitoring. Besides, they are also linked to an active referral system, so they know where women may get higher-level care in the event of an obstetric emergency. Furthermore, as midwives can be trained and supported at a cheap cost, and because their pay are significantly lower than those of medical doctors, effective utilization of this group of health professionals is one of the keys to saving mothers’ lives on a limited budget [139]. Similar approach is observed in Maldives, another example of highest declining country in maternal mortality. Primarily, Maldives has prioritized investments and policies aimed at maternal and child health. Since 1991, the number of nurses and midwives in the population has increased by a factor of twelve which is four times more than the average in South-Asia [140].

#### Strategies to improve.

While household economic status appeared to play a role in the inequality in health-care utilization, women’s educational attainment can also help to narrow the non-poor/poor gap in MHC access as they access the care more if they are aware of it [53,141]. Development and implementation of interventions, such as community awareness campaigns, to change the behavior of husbands and/or mothers-in-law in order to persuade pregnant women to access ANC, to reduce the cost of travel or to improve transportation infrastructure, as well as providing free or subsidized medicines is also necessary. Through a public-private partnership, the proposed interventions can be tested in community-based trials in various regions around the country [100]. Strong frontline workers such as trained midwives can provide more service in rural areas and if they have proper training, they can understand danger signs early. Healthcare reform should also reconsider both the demand (committing to proper risk-pooling mechanisms for the entire population, expanding benefits, and lowering cost-sharing) and supply sides of healthcare reform should be reconsidered (expansion of infrastructure, human resources for health, and health services) [24]. There are some health-care intervention delivery programs used in low-middle income countries are proved to be successful, such as, national health insurance scheme, conditional cash transfer, voucher scheme, community-based health insurance scheme, alternate transport system, health literacy through women’s group etc. [23,142–145]. Any intervention taken for fighting inequalities in access to MHC must be adaptive to the context.

#### Strength and limitations.

A key strength of this study is that, by far, it is the first systematic review to comprehensively analyze socioeconomic inequalities in accessing MHC in South Asia. This contributes to the existing literature by accumulating evidence across multiple countries. While quantitative findings are generalizable due to large-scale survey data, qualitative studies provide valuable, in-depth insights into the social and structural barriers faced by women due to their socio-economic position. Although these studies were conducted in specific regions, their strength lies in capturing the complexities of real-life experiences, which may not always be reflected in large-scale surveys.

This systematic analysis can also help public health professionals and policymakers better tailor health services to the needs of mothers from low socioeconomic position and eliminate health inequalities. However, this study also has some limitations as well. Such as, search strategy was limited to only two databases. Papers were also extracted from the references. There is still a possibility that all relevant papers are not included. Besides, studies from other language than English were missed. Some studies did not adjust for potential confounders or did not mention about it. Those who did adjusted for a wide range of confounders. A notable variation in measures of socioeconomic factors were observed. Education level and women autonomy measure was also defined in various ways in different papers. As majority of the studies were cross-sectional, therefore causal relationship could not be established. Also, these studies used self-reported answers from mother who gave birth in last 5 years, which may lead to recall bias. Moreover, this review excluded studies on indigenous, tribal, and migratory populations. While this exclusion demonstrates a more focused analysis, it may limit the generalizability of the findings to these vulnerable groups.

### Conclusion

Systematic review indicates there are significant socioeconomic inequalities in every component of MHC in most of the South-Asian countries. However, to understand the causal relationship and actual magnitude of the problem, further studies are needed such as, meta-analysis or large control group interventions. Socioeconomic inequalities in access to maternal healthcare is a multidimensional issue. Especially, in South-Asian region, this problem has complex and divergent nature due to multicultural structure. Therefore, it is crucial to apprehend the extent of the problem to implement country and context specific approach. This study will create a baseline for future research in understanding in-depth about the inequality to MHC access.

### Supporting information

S1 File Search strategy. (DOCX)

S2 File Quality assessment. (XLSX)

S3 File PRISMA_2020_checklist. (DOCX)

S4 File S4–S9 Tables: Detailed result of each paper. (DOCX)

S1 Table All studies. (XLSX)

S2 Table Included studies. (XLSX)
