# Supplementary material for: Socioeconomic inequalities in access to maternal healthcare in South-Asian countries: A systematic review
Source: PLoS One. 2025 Jun 17;20(6):e0326130. doi: 10.1371/journal.pone.0326130 (PMC12173378; doi:10.1371/journal.pone.0326130)
Supplement: S1 File — (DOCX) [file pone.0326130.s001.docx]

**Supplementary material 1 (S1) Search code for MEDLINE through Pubmed**

(“Socioeconomic factors”[MeSH] OR “socio*”[tiab] OR “SES”[tiab] OR “occupation”[tiab] OR “income”[tiab] OR “employment”[tiab] OR “education”[tiab] OR “wealth”[tiab] OR “rural”[tiab] OR “cost”[tiab] OR “poverty”[tiab] OR “household”[tiab]) AND (“Healthcare disparities”[MeSH] OR “Inequality”[tiab] OR “access”[tiab] OR “accessibility”[tiab] OR “affordability”[tiab] OR “availability”[tiab] OR “acceptance”[tiab] OR “accommodation”[tiab] OR “inequity”[tiab] OR “unmet needs”[tiab] OR “barrier”[tiab] OR “difficult”[tiab] OR “challenge”[tiab] OR “obstacle”[tiab] OR “problem”[tiab] OR “stigma”[tiab] OR “transport”[tiab]) AND (“Maternal health”[tiab] OR “maternal health services”[MeSH] OR “maternity”[tiab] OR “prenatal”[tiab] OR “postpartum”[tiab] OR “perinatal”[tiab] OR “antenatal”[tiab] OR “postnatal”[tiab] OR “obstetric”[tiab] OR “emergency”[tiab] OR “reproductive health”[tiab] OR “ANC”[tiab] OR “pregnancy”[tiab] OR “birth”[tiab] OR “labor”[tiab] OR “labour”[tiab] OR “skilled delivery”[tiab] OR “c-section”[tiab] OR “caesarian section”[tiab]) AND (“Southern Asia”[tiab] OR “South-Asia”[tiab] OR “Bangladesh” OR “India” OR “Pakistan” OR “Afghanistan” OR “Maldives”[tiab] OR “Republic of Maldives”[tiab] OR “Sri Lanka” OR “Bhutan” OR “Nepal”)

**Supplementary table (S2): Quality assessment score and contextual issue**

| Cross sectional analytical study | | | | |
| --- | --- | --- | --- | --- |
|  | Author | Study | MMAT assessment | Contextual issue |
| 1 | Chalise B, 2019 | Correlates of continuum of maternal health services among Nepalese women: Evidence from Nepal Multiple Indicator Cluster Survey | 100% (good) | - |
| 2 | Azimi MW, 2019 | Factors associated with antenatal care visits in Afghanistan: secondary analysis of Afghanistan Demographic and Health Survey 2015 | 80% (moderate) | - Women who did not know about ANC was excluded from the study, however, this can also be an important factor related with education |
| 3 | Khatiwada J, 2020 | Dimensions of women's empowerment on access to skilled delivery services in Nepal. | 100% (good) | - |
| 4 | Singh L, 2019 | Measuring quality of antenatal care: a secondary analysis of national survey data from India. | 100% (good) | - Even though data was extracted from demographic survey data, it is limited to district level data whereas, effect of socio-economic factors are more prominent in villages |
| 5 | Bhowmik J, 2019 | Antenatal care and skilled birth attendance in Bangladesh are influenced by female education and family affordability: BDHS 2014 | 100% (good) | - |
| 6 | Yaya S, 2017 | Factors associated with the utilization of institutional delivery services in Bangladesh. | 100% (good) | - |
| 7 | Mumtaz S, 2019 | Current status and determinants of maternal healthcare utilization in Afghanistan: Analysis from Afghanistan Demographic and Health Survey 2015. | 100% (good) | - |
| 8 | Dhakal S, 2011 | Skilled care at birth among rural women in Nepal: practice and challenges. | 100% (good) | - Sample size is small, only 150 women to draw out a strong association |
| 9 | Chanda SK, 2019 | Factors associating different antenatal care contacts of women: A cross-sectional analysis of Bangladesh demographic and health survey 2014 data. | 100% (good) | - May have subjected to recall bias - Small spatial units like subdistrict, village is not addressed which is also socio-economically disadvantaged - Accessibility of the ANC is explored without addressing the availability of the resource |
| 10 | Zere E, 2013 | Equity in reproductive and maternal health services in Bangladesh | 80% (moderate) | - Can’t tell if confounders are taken into consideration or not - The paper focuses on women both on reproductive and obstetric care, therefore, keeping a big age limit starting from 10 to 49 |
| 11 | Pallikadavath S, 2004 | Antenatal care: provision and inequality in rural north India. | 100% (good) | - |
| 12 | Singh A, 2012 | Socio-economic inequalities in the use of postnatal care in India. | 100% (good) | - |
| 13 | Mistry R, 2009 | Women’s autonomy and pregnancy care in rural India: a contextual analysis | 100% (good) | - |
| 14 | Pulok MH, 2016 | Progress in the utilization of antenatal and delivery care services in Bangladesh: where does the equity gap lie? | 100% (good) | - |
| 15 | Dhakal S, 2007 | Utilisation of postnatal care among rural women in Nepal | 60% (poor) | - There was chance of missing out eligible women thus, it is unsure if there is complete outcome data - Variables are not explained properly and not justified how they were calculated in the statistical tests - Adjustment for confounders are not mentioned - They mention women’s education is significant with formal sector job but they did not provide the quantitative finding in the result section |
| 16 | Prakash R, 2013 | Urban poverty and utilization of maternal and child health care services in India. | 100% (good) | - |
| 17 | Jain AK, 2015 | The constraints of distance and poverty on institutional deliveries in Pakistan: evidence from georeferenced-linked data | 100% (good) | - |
| 18 | Kamal SM, 2015 | Inequality of the use of skilled birth assistance among rural women in Bangladesh: facts and factors | 100% (good) | - |
| 19 | Bhatta DN, 2015 | Paternal Factors and Inequity Associated with Access to Maternal Health Care Service Utilization in Nepal: A Community Based Cross-Sectional Study | 100% (good) | - |
| 20 | Khanal V, 2014 | Factors associated with the utilization of postnatal care services among the mothers of Nepal: analysis of Nepal demographic and health survey 2011 | 100% (good) | - |
| 21 | Ghaffar A, 2015 | Factors associated with utilization of antenatal care services in Balochistan province of Pakistan: An analysis of the Multiple Indicator Cluster Survey (MICS) 2010 | 80% (moderate) | - Can’t tell if confounders are taken into account or not |
| 22 | Dalal K, 2012 | Economic empowerment of women and utilization of maternal delivery care in Bangladesh | 100% (good) | - |
| 23 | Sahito A, 2018 | Inequities in Antenatal Care, and Individual and Environmental Determinants of Utilization at National and Sub-national Level in Pakistan: A Multilevel Analysis | 100% (good) | - |
| 24 | Huda TM, 2019 | Individual and community level factors associated with health facility delivery: A cross sectional multilevel analysis in Bangladesh | 100% (good) | - |
| 25 | Kc S, 2016 | Women’s Autonomy and Skilled Attendance During Pregnancy and Delivery in Nepal | 100% (good) | - |
| 26 | Sridharan S, 2017 | Towards an understanding of the multilevel factors associated with maternal health care utilization in Uttar Pradesh, India | 100% (good) | - |
| 27 | Krishnamoorthy Y, 2020 | Equity in coverage of maternal and newborn care in India: evidence from a nationally representative survey | 100% (good) | - |
| 28 | Zuhair M, 2017 | Socioeconomic Determinants of the Utilization of Antenatal Care and Child Vaccination in India | 100% (good) | - |
| 29 | Pulok MH, 2018 | Socioeconomic inequality in maternal healthcare: An analysis of regional variation in Bangladesh | 100% (good) | - |
| 30 | Akseer N, 2016 | Coverage and inequalities in maternal and child health interventions in Afghanistan | 100% (good) | - |
| 31 | Budhwani H, 2015 | Individual and Area Level Factors Associated with Prenatal, Delivery, and Postnatal Care in Pakistan | 100% (good) | - |
| 32 | Awasthi A, 2016 | Disparity in maternal, newborn and child health services in high focus states in India: a district-level cross-sectional analysis | 100% (good) | - All districts are not included, due to vast diversity in Indian population, result cannot be generalized |
| 33 | Joshi C, 2014 | Factors associated with the use and quality of antenatal care in Nepal: a population-based study using the demographic and health survey data | 100% (good) | - |
| 34 | Rahman MA, 2021 | Factors influencing place of delivery: Evidence from three south-Asian countries | 100% | - |
| 35 | Yeo S, 2022 | Afghan women’s empowerment and antenatal care utilization: a population-based cross-sectional study. | 100% | - |
| 36 | Methun MIH, 2022 | Socioeconomic correlates of Adequate Maternal Care in Bangladesh: Analysis of the Bangladesh Demographic and Health Survey 2017-18 | 100% | - |
| 37 | Thapa B, 2023 | Determinants of institutional delivery service utilization in Nepal | 100% | - |
| 38 | StanikzaiM.H., 2023 | Contents of antenatal care services in Afghanistan: findings from the national health survey 2018 | 100% |  |
| 39 | Thakkar N, 2023 | Factors associated with underutilization of antenatal care in India: Results from 2019–2021 National Family Health Survey | 100% | - |
| 40 | Jannat Z, 2023 | Factors affecting practices of recently delivered women on maternal and neonatal health care in selected rural areas of Bangladesh | 80% | - Small-scale study conducted in only two unions of Bangladesh and thus, the findings cannot be generalized. - Although total sample size was 550 but for ANC and PNC visits it was 245 and 133, respectively. |
| 41 | Misu F, 2023 | Comparison of inequality in utilization of postnatal care services between Bangladesh and Pakistan: Evidence from the Demographic and Health Survey 2017-2018 | 100% | - |
| 42 | Misu F, 2023 | Comparison of inequality in utilization of maternal healthcare services between Bangladesh and Pakistan: evidence from the demographic health survey 2017-2018 | 100% | - |
| 43 | Methun MIH, 2023 | Inequalities in adequate maternal healthcare opportunities: evidence from Bangladesh Demographic and Health Survey 2017-2018 | 100% | - |
| Qualitative study | | | | |
| 44 | Vidler M, 2016 | Utilization of maternal health care services and their determinants in Karnataka State, India | 100% (good) | - |
| 45 | Afsana K, 2004 | The tremendous cost of seeking hospital obstetric care in Bangladesh | 60% (poor) | - Details about the analysis was not mentioned |
| 46 | Lama S, 2014 | Barriers in Utilization of Maternal Health Care Services: Perceptions of Rural Women in Eastern Nepal | 60% (poor) | - How many women were included in each focus group, number of all participants were not mentioned - By maternal healthcare, what type of care is indicated in the interview was not explained |
| 47 | Rahmani Z, 2013 | Antenatal and obstetric care in Afghanistan--a qualitative study among health care receivers and health care providers | 100% (good) | - |
| 48 | Nisar YB | Qualitative exploration of facilitating factors and barriers to use of antenatal care services by pregnant women in urban and rural settings in Pakistan | 100% (good) | - |
| 49 | Milne L, 2015 | Staff perspectives of barriers to women accessing birthing services in Nepal: a qualitative study | 100% (good) | - |
| 50 | Mumtaz Z, 2014 | Improving maternal health in Pakistan: toward a deeper understanding of the social determinants of poor women's access to maternal health services | 100% (good) | - |
| 51 | Pitchforth E, 2006 | Getting women to hospital is not enough: a qualitative study of access to emergency obstetric care in Bangladesh | 100% (good) | - |
| 52 | Mahapatro M, 2015 | Equity in utilization of health care services: Perspective of pregnant women in southern Odisha, India | 100% (good) | - |
| 53 | Memon Z, 2015 | Residual Barriers for Utilization of Maternal and Child Health Services: Community Perceptions from Rural Pakistan | 100% (good) | - |
| Mixed Method Study | | | | |
| 54 | Higgins-Steele A, 2018 | Barriers associated with care-seeking for institutional delivery among rural women in three provinces in Afghanistan | 60% (poor) | - Questionnaire for collecting data was not pre-tested - Qualitative part of the study is not well explained, it does explain the reasons or mindset behind quantitative findings but not brief |
| 55 | Ansari MS, 2015 | Access to comprehensive emergency obstetric and newborn care facilities in three rural districts of Sindh province, Pakistan | 80% (moderate) | - It only mentioned descriptive statistic are used, however, no further explanation is given on what kind of analysis was done |
