# Supplementary material for: Socioeconomic inequalities in access to maternal healthcare in South-Asian countries: A systematic review
Source: PLoS One. 2025 Jun 17;20(6):e0326130. doi: 10.1371/journal.pone.0326130 (PMC12173378; doi:10.1371/journal.pone.0326130)
Supplement: S4 File — (DOCX) [file pone.0326130.s004.docx]

**Supplementary Table (S4): Quantitative results by economy**

| Study | Indicator | Sample size | | Quantitative findings | | | | | | | | | | | | | |
| --- | --- | --- | --- | --- | --- | --- | --- | --- | --- | --- | --- | --- | --- | --- | --- | --- | --- |
| **Country: India** | | | | | | | | | | | | | | | | | |
| Singh L, 2019 | ANC | 190898 | | OR, 95% CI, *p value significant at <0.001 | | | | | | | | | | | | | |
|  |  |  |  | Wealth Quintile | | | | No & inadequate ANC vs adequate  ANC | | | | | | | | | |
|  |  |  |  | Poorest  Poorer  Middle  Richer  Richest | | | | Ref.  1.46 (1.36, 1.57)*  1.80 (1.67, 1.94)*  1.98 (1.82, 2.15)*  2.73 (2.47, 3.01)* | | | | | | | | | |
| Pallikadavath S, 2004 | ANC | 11369 | | OR, 95% CI, ** p < 0.010; * p < 0.05 | | | | | | | | | | | | | |
|  |  |  |  | State | | | | Standard of living | | | | | | | | | |
|  |  |  |  |  |  |  |  | With home visit | | | | Without home visit | | | | | |
|  |  |  |  | Bihar | | | | 1.3** | | | | 1.2 | | | | | |
|  |  |  |  | Madhya Pradesh | | | | 1.3* | | | | 1.2 | | | | | |
|  |  |  |  | Rajasthan | | | | 1.1 | | | | 1.2 | | | | | |
|  |  |  |  | Uttar Pradesh | | | | 1.2** | | | | 1.3*** | | | | | |
| Singh A, 2012 | ANC & PNC (Postnatal) | 216831 | | Rich-poor ratio; *** p < 0.0001 | | | | | | | | | | | | | |
|  |  |  |  | 1.7*** (any ANC)  3.4*** (4+ ANC)  3.4*** (PNC; within 48h) | | | | | | | | | | | | | |
|  |  |  |  | Concentration index | | | | | | | | | | | | | |
|  |  |  |  | 0.074*** (PNC; within 48h, maternal complication) | | | | | | | | | | | | | |
| Prakash R, 2013 | ANC, SD | 82837 | | Effect of poverty on ANC and SD, Predicted probability, 95% CI | | | | | | | | | | | | | |
|  |  |  |  | Country/ district | | | | ANC | | | | SD | | | | | |
|  |  |  |  | India | | | | 0.44 (Poor, p < 0.001)  0.72 (Non-poor, p < 0.001) | | | | 0.52 (Poor, p < 0.001)  0.90 (Non-poor, p <0.001) | | | | | |
|  |  |  |  | Uttaranchal | | | | - | | | | 0.11 (Poor, p < 0.001)  0.91 (Non-poor, p < 0.001) | | | | | |
|  |  |  |  | Rajasthan | | | | 0.33 (Poor, p < 0.001)  0.78 (Non-poor, p < 0.001) | | | | 0.47 (Poor, p < 0.001)  0.92 (Non-poor, p < 0.001) | | | | | |
|  |  |  |  | Uttar Pradesh | | | | 0.20 (Poor, p < 0.012)  0.46 (Non-poor, p < 0.012) | | | | 0.19 (Poor, p < 0.001)  0.68 (Non-poor, p < 0.001) | | | | | |
|  |  |  |  | Madhya Pradesh | | | | 0.31 (Poor, p < 0.001)  0.74 (Non-poor, p < 0.001) | | | | 0.30 (Poor, p < 0.001)  0.91 (Non-poor, p < 0.001) | | | | | |
|  |  |  |  | Maharashtra | | | | 0.44 (Poor, p < 0.001)  0.72 (Non-poor, p < 0.001) | | | | 0.52 (Poor, p < 0.001)  0.90 (Non-poor, p < 0.001) | | | | | |
|  |  |  |  | Tamilnadu | | | | 0.44 (Poor, p < 0.001)  0.72 (Non-poor, p < 0.001) | | | | 0.52 (Poor, p < 0.001)  0.90 (Non-poor, p < 0.001) | | | | | |
| Sridharan S 2017 | ANC | 5666 | | OR, 95% CI, ***p < 0.01; **p < 0.05. | | | | | | | | | | | | | |
|  |  |  |  | Uttar Pradesh | | | | | | | | | | | | | |
|  |  |  |  | 2.83*** (Own house)  1.11*** (Wealth index) | | | | | | | | | | | | | |
| Krishnamoorthy Y, 2020 | ANC, ID, PNC | 190797 | | Rich-poor ratio & | | | | | | Concentration index | | | | | | | |
|  |  |  |  | *P < 0.001 | | | | | |  | | | | | | | |
|  |  |  |  | 2.89 (4 +ANC visit)  1.56 (ID)  1.68 (PNC 24h)  1.66 (PNC 8h) | | | | | | 0.195* (4+ ANC visit)  0.090* (ID)  0.106* (PNC 24h)  0.103* (PNC 8h) | | | | | | | |
|  |  |  |  | 4+ ANC | | | | | | | | | | | | | |
|  |  |  |  | Region | | | | | | Concentration index (significant at least 0.2) | | | | | | | |
|  |  |  |  | North  Central  East  North-east  West  South | | | | | | 0.176  0.263  0.227  0.161  0.066  0.029 | | | | | | | |
| Zuhair M, 2017 | ANC | 36447 | | Wealth quintile; Richest (Ref.) | | | | | | *p < 0.05 | | | | | | | |
|  |  |  |  | Richer  Middle  Poorer  Poorest | | | | | | 0.51*  0.37*  0.23*  0.15* | | | | | | | |
| Awasthi A, 2016 | ANC, SBA | 3104 | | Coverage gap (Wealth quintile) | | | | | | | | | | | | | |
|  |  |  |  | 19.52 (ANC)  14.50 (SBA) | | | | | | | | | | | | | |
| Thakkar N, 2023 | ANC | 172702 | | Household wealth index, AOR, 95% CI, p-value significant at <0.05 | | | | | | | | | | | | | |
|  |  |  |  | Wealth index | | | | | | Inadequate ANC visits | | | | | | | p-value |
|  |  |  |  | Poorest  Poorer  Middle  Richer  Richest | | | | | | 1.69 (1.57–1.81)  1.33 (1.24–1.42)  1.16 (1.09–1.23)  1.09 (1.02–1.16)  Ref. | | | | | | | <0.001  <0.001  <0.001  0.011  - |
|  | | | **Country: Nepal** | | | | | | | | | | | | | | |
| Chalise B, 2019 | ANC, SBA, PNC | 2086 | | AOR; 95% CI; *p < 0.05 | | | | | | | | | | | | | |
|  |  |  |  | Wealth quintile | | | | ANC | | ANC, SBA | | | | | ANC, SBA, PNC | | |
|  |  |  |  | Reference (low) | | | | Ref. | | Ref. | | | | | Ref. | | |
|  |  |  |  | Middle | | | | 2.34* (1.59, 3.44) | | 2.36* (1.60, 3.54) | | | | | 2.56* (1.68, 3.91) | | |
|  |  |  |  | Rich | | | | 3.83* (2.49, 5.88) | | 3.97* (2.69, 5.85) | | | | | 4.50* (3.07, 6.59) | | |
| Dhakal S, 2011 | Skilled delivery | 150 | | Not utilizing due to lack of money | | | | | | | | | | | | | |
|  |  |  |  | 21.7% (Hospital)  14.4% (Home) | | | | | | | | | | | | | |
| Bhatta DN, 2015 | ANC, ID | 2178 | | AOR; 95% CI; p < 0.001 | | | | | | | | | | | | | |
|  |  |  |  | Wealth Quintile (Paternal) | | | | ANC | | | | ID | | | | | |
|  |  |  |  | Poorest  Richest | | | | Ref.  1.99 | | | | Ref.  2.74 | | | | | |
|  |  |  |  | Concentration index score | | | | | | | | | | | | | |
|  |  |  |  | 0.05 (ID)  0.04 (ANC)  0.05 (SBA) | | | | | | | | | | | | | |
| Khanal V, 2014 | PNC | 4079 | | AOR, 95% CI; p < 0.002 | | | | | | | | | | | | | |
|  |  |  |  | Reference (Poor; lower 40%)  1.638 (Middle; middle 40%)  3.182 (Rich; upper 20%) | | | | | | | | | | | | | |
| Joshi C, 2014 | ANC | 4079 | | Wealth index | | | | | | AOR, 95% CI; p < 0.001 | | | | | | | |
|  |  |  |  | Poorest  Poorer  Middle  Richer  Richest | | | | | | Ref.  1.17 (0.88, 1.56)  1.28 (0.90, 1.83)  1.87 (1.28, 2.74)  3.00 (1.95, 4.60) | | | | | | | |
| Rahman MA 2021 | Facility based delivery | 16,429 (3962 in Nepal) | | Household Wealth Quantile; AOR, 95% CI; *p<0.05, **p < 0.01, ***p < 0.001 | | | | | | | | | | | | | |
|  |  |  |  | Poor  Middle  Rich | | | | | | Ref.  1.61(1.31–1.98) ***  2.32(1.88–2.86) *** | | | | | | | |
| Thapa B, 2023 | ID | 1932 | | Household Wealth Quantile; AOR, 95% CI; *p<0.05, **p < 0.01, ***p < 0.001, AIC: Akaike Information Criterion (Best Fitting Model, Final Model) | | | | | | | | | | | | | |
|  |  |  |  | Poorest  Second  Middle  Fourth  Richest | | | | | | 0.09(0.04–0.22) ***  0.29(0.13–0.64) **  0.48(0.21–1.08)  0.65(0.28–1.46)  Ref. | | | | | | | |
|  |  |  |  | AIC | | | | | | 1484.94 | | | | | | | |
| **Country: Bangladesh** | | | | | | | | | | | | | | | | | |
| Pulok MH, 2018 | ANC, SBA | 4483 | | Region*wealth | | | | | | OR, 95% CI; *p < 0.1, **p < 0.05, ***p < 0.01 | | | | | | | |
|  |  |  |  | Rangpur  Barishal  Chittagong  Dhaka  Khulna  Rajshahi  Sylhet | | | | | | Ref.  1.24***  1.72***  1.21***  1.29***  1.36***  1.33*** | | | | | | | |
|  |  |  |  | Erreygers Index, 95% CI | | | | | | | | | | | | | |
|  |  |  |  | Region | | | | 1 ANC | | | | 4+ ANC | | | | | |
|  |  |  |  | Barishal  Chittagong  Dhaka  Khulna  Rajshahi  Khulna  Sylhet | | | | 0.289  0.471  0.241  0.170  0.352  0.132  0.446 | | | | 0.237  0.355  0.385  0.311  0.363  0.085  0.313 | | | | | |
|  |  |  |  |  | | | | Health facility delivery | | | | Medically trained delivery | | | | | |
|  |  |  |  | Barishal  Chittagong  Dhaka  Khulna  Rajshahi  Khulna  Sylhet | | | | 0.505  0.478  0.524  0.437  0.461  0.280  0.370 | | | | 0.530  0.462  0.510  0.408  0.455  0.323  0.422 | | | | | |
| Huda TM, 2019 | Health facility delivery/ ID | 28032 | | Wealth index | | | | | | AOR, 95% CI | | | | | | | |
|  |  |  |  | Poorest  Second  Middle  Fourth  Richest | | | | | | Ref.  1.13 (0.99,130)  1.53 (1.34,1.74)  1.79 (1.57, 2.04)  3.15 (2.72,3.65) | | | | | | | |
| Dalal K, 2012 | ID | 4925 | | AOR, 95% CI; ***p < 0.001, **p < 0.010, *p < 0.05 | | | | | | | | | | | | | |
|  |  |  |  | Home delivery by economic status | | | | | | | | | | | | | |
|  |  |  |  | Poorest  Poorer  Middle  Richer  Richest | | | | | | 7.26 (2.19–24.03)**  7.05 (2.22–22.36)**  6.34 (2.07–19.40)**  2.91 (1.23–6.88)*  Ref. | | | | | | | |
| Kamal SM, 2015 | SBA | 4809 | | AOR, 95% CI; p *<0.05, **<0.01, ***<0.001 | | | | | | | | | | | | | |
|  |  |  |  | Poorest  Poorer  Middle  Richer  Richest | | | | | | Ref.  1.04 (0.71-1.53)*  1.50 (1.04-2.18)*  2.16 (1.50-3.10)***  5.11 (3.40-7.68)*** | | | | | | | |
| Pulok MH, 2016 | ANC, SBA, ID | 3730 (2004)  3365 (2007)  4648 (2011) | | OR, 95% CI; *p <0.1, **p < 0.05, ***p < 0.001 | | | | | | | | | | | | | |
|  |  |  |  | Wealth quintile | | | | 4+ANC | | ID | | | | | SBA | | |
|  |  |  |  | Poorest  Poorer  Middle  Richer  Richest | | | | Ref.  1.00  1.24  1.70***  2.91*** | | Ref.  0.92  1.18  1.82***  3.16*** | | | | | Ref.  0.87  1.30  1.82***  3.32*** | | |
|  |  |  |  | Association with microfinance program | | | | | | | | | | | | | |
|  |  |  |  | No | | | | Ref. | | Ref. | | | | | Ref. | | |
|  |  |  |  | Yes | | | | 1.22** | | 1.10 | | | | | 1.18 | | |
| Zere E, 2013 | ANC, SBA, ID | 11178 | | Indicator | | | | Relative index of inequality (RII) | | Lower CI | | | | | Upper CI | | |
|  |  |  |  | 4 ANC  SBA delivery  Doc. Delivery  Nurse/midwife delivery  ID | | | | 2.5  3.7  3.6  3.3  3.3 | | 1.5  1.5  1.7  1.7  1.6 | | | | | 3.6  5.9  5.5  4.9  5.0 | | |
| Chanda SK, 2020 | ANC | 4475 | | OR, 95% CI | | | | | | | | | | | | | |
|  |  |  |  | Wealth index | | | | ANC contact | | | | 8+ ANC | | | | | |
|  |  |  |  | Richest  Richer  Middle  Poorer  Poorest | | | | 4.85 (3.15, 7.47)  2.47 (1.85, 3.31)  1.63 (1.27, 2.08)  1.29 (1.05, 1.59)  Ref. | | | | 1.86 (1.02, 3.39)  1.30 (0.73, 2.33)  1.08 (0.60, 1.96)  1.55 (0.88, 2.73)  Ref. | | | | | |
| Yaya S, 2017 | ID | 7313 | | Household wealth | | | | OR, 95% CI (Conditional logistic regression); ***p < 0.05 | | | | | | | | | |
|  |  |  |  | Poor  Average  Rich | | | | Ref.  1.272 (1.057, 1.531)***  2.507 (2.118, 2.968)*** | | | | | | | | | |
| Bhowmik J, 2019 | ANC, SBA | 17863 | | OR, 95% CI; Mixed effect model | | | | | | | | | | | | | |
|  |  |  |  | Wealth index | | | | ANC | | | | SBA | | | | | |
|  |  |  |  | Poorest  Poorer  Middle  Richer  Richest | | | | Ref.  1.52 (1.21, 1.90)  2.04 (1.59, 2.60)  2.61 (1.98, 3.42)  5.08 (3.50, 7.38) | | | | Ref.  1.47 (1.41, 1.91)  1.91 (1.47, 2.49)  2.46 (1.87, 3.24)  4.31 (3.31, 5.93) | | | | | |
| Rahman MA 2021 | Facility based delivery | 16,429 (4278 in Bangladesh) | | Household Wealth Quantile; AOR, 95% CI; *p<0.05, **p < 0.01, ***p < 0.001 | | | | | | | | | | | | | |
|  |  |  |  | Poor  Middle  Rich | | | | | | Ref.  1.23(0.99–1.52)  1.66(1.34–2.07) *** | | | | | | | |
| Methun MIH 2022 | ANC, ID | 4012 | | Wealth status, OR, 95% CI, P-value<0.05*, P-value<0.01**, P-value<0.001*** | | | | | | | | | | | | | |
|  |  |  |  | Richest  Richer  Middle  Poorer  Poorest | | | | | | Ref.  0.491 (0.402, 0.601)***  0.414 (0.329, 0.52)***  0.218 (0.167, 0.285)***  0.170 (0.127, 0.228)*** | | | | | | | |
| Misu F, 2023 | PNC | Bangladesh (4440) | | Wealth status, RCI: Relative Concentration Index; ACI: Absolute Concentration Index; SII: Slope Index of inequality; CI: Confidence interval, significant at p≤0.05 | | | | | | | | | | | | | |
|  |  |  |  | Wealth status | | | | PNC Check of Women within 2 Days by Skilled Provider | | | | | | | | | |
|  |  |  |  | RCI [95% CI]  ACI [95% CI]  SII [95% CI] | | | | 0.448 [0.413–0.483], p-value = 0.00  0.448 [0.413–0.483], p-value = 0.00  0.643 [0.600-0.685], p-value = 0.00 | | | | | | | | | |
| Misu F, 2023 | ANC, SBA, ID | Bangladesh (4948) | | Wealth status, RCI: Relative Concentration Index; ACI: Absolute Concentration Index; SII: Slope Index of inequality, Standard error is in parenthesis. | | | | | | | | | | | | | |
|  |  |  |  | Wealth Status | | | | At least four  ANC visits  by skilled  provider | | Skilled Birth  Attendance (SBA) | | | | Facility-based  Delivery | | | |
|  |  |  |  | RCI  ACI  SII | | | | 0.311 (0.02)  0.310 (0.02)  0.461 (0.03) | | 0.424 (0.02)  0.423 (0.02)  0.612 (0.02) | | | | 0.403 (0.02)  0.403 (0.02)  0.586 (0.02) | | | |
| Methun MIH, 2023 | ANC, ID | Bangladesh (5012) | | Shapley’s decomposition method for the relative contribution of wealth status to the  Inequality | | | | | | | | | | | | | |
|  |  |  |  |  | | | | Access to minimum  required ANC visits (4+ANC) | | | | Access to institutional  Delivery | | | | | |
|  |  |  |  | Wealth Status | | | | 39.7% | | | | 43.4% | | | | | |
| **Country: Pakistan** | | | | | | | | | | | | | | | | | |
| Jain AK, 2015 | ID | 4435 | | AOR, 95% CI; *p < .05; **p < .01; ***p < .001. | | | | | | | | | | | | | |
|  |  |  |  | Household wealth | | | | Net effect | | | | Individual effect | | | | | |
|  |  |  |  | Lowest  Lower middle  Upper middle  Highest | | | | Ref.  1.33*  1.80***  3.13*** | | | | Ref.  1.36*  1.83***  3.19*** | | | | | |
| Ghaffar A, 2015 | ANC | 2339 | | Akaike Information Criterion; p value = *0.1, **0.05, ***0.01 | | | | | | | | | | | | | |
|  |  |  |  | Factor | | | | Estimate | | | | Exp (estimate) | | | | | |
|  |  |  |  | Wealth index score | | | | 0.547*** | | | | 1.72 | | | | | |
| Sahito A, 2018 | ANC | 7142 | | Wealth index, OR, 95% CI | | | | | | | | | | | | | |
|  |  |  |  | Baluchistan (BC), Gilgit Baltistan (GB), Khyber Pakhtunkhwa (KPK) | | | | | | | | | | | | | |
|  |  |  |  | Level | BC | | | GB | | KPK | | | Sindh | | | Punjab | |
|  |  |  |  | Poorest  Poorer  Middle  Richer  Richest | Ref.  1.5 (0.7, 3.3)  2.1 (0.7, 6.3)  1.5 (0.6, 3.6)  3.8 (0.9, 15.0) | | | Ref.  2.1 (1.1, 3.8)  1.4 (0.7, 3.0)  4.8 (1.9, 11.9)  3.5 (0.3, 36.1) | | Ref.  1.7 (0.9, 3.2)  2.0 (1.0, 4.0)  3.3 (1.5, 7.1)  7.7 (3.6, 16.5) | | | Ref.  1.5 (1.0, 2.4)  2.3 (1.5, 3.5)  3.3 (2.1, 5.3)  5.8 (3.2, 10.7) | | | Ref.  1.6 (0.9, 2.8)  2.1 (1.2, 3.7)  3.2 (1.8, 5.7)  6.0 (3.3, 11.0) | |
| Budhwani H, 2015 | ANC, ID, SBA, PNC | 7399 | | OR, 95% CI; *p < 0.05, **p < 0.01, ***p < 0.001 | | | | | | | | | | | | | |
|  |  |  |  | Factor | | 4+ ANC | | | ID | | SBA | | | PNC | | | |
|  |  |  |  | Household wealth | | 1.607*** | | | 1.580*** | | 1.548*** | | | 1.401* | | | |
| Ansari MS, 2015 | EmONC | 690 (Household survey);  309 (Exit survey) | | Unaffordable travel cost to health facility | | | | Household survey | | | | | | | | | |
|  |  |  |  |  |  |  |  | Within 5 kilometers | | | | More than 5 kilometers | | | | | |
|  |  |  |  | Public | | | | 49% | | | | 63% | | | | | |
|  |  |  |  | Private | | | | 73% | | | | 70% | | | | | |
| Rahman MA 2021 | ID | 16,429 (8189 in Pakistan) | | Household Wealth Quintile; AOR, 95% CI; p < 0.001 | | | | | | | | | | | | | |
|  |  |  |  | Poor  Middle  Rich | | | | | | Ref.  1.36(1.17–1.59) ***  1.83(1.54–2.18) *** | | | | | | | |
| Misu F, 2023 | PNC | Pakistan (3780) | | RCI: Relative Concentration Index; ACI: Absolute Concentration Index; SII: Slope Index of inequality; CI: Confidence interval, significant at p≤0.05 | | | | | | | | | | | | | |
|  |  |  |  | Wealth status | | | | PNC Check of Women within 2 Days by Skilled Provider | | | | | | | | | |
|  |  |  |  | RCI [95% CI]  ACI [95% CI]  SII [95% CI] | | | | 0.397 [0.332–0.462], p-value = 0.00  0.397 [0.331–0.462], p-value = 0.00  0.598 [0.514–0.681], p-value = 0.00 | | | | | | | | | |
| Misu F, 2023 | ANC, SBA, ID | Pakistan (5122) | | Wealth status, RCI: Relative Concentration Index; ACI: Absolute Concentration Index; SII: Slope Index of inequality, Standard error is in parenthesis. | | | | | | | | | | | | | |
|  |  |  |  | Wealth Status | | | At least four ANC visits by skilled provider | | | Skilled Birth Attendance (SBA) | | | | | Facility-Based Delivery | | |
|  |  |  |  | RCI  ACI  SII | | | 0.516 (0.02)  0.516 (0.02)  0.738 (0.02) | | | 0.459 (0.03)  0.358 (0.03)  0.569 (0.04) | | | | | 0.451 (0.03)  0.376 (0.03)  0.587 (0.04) | | |
| **Country: Afghanistan** | | | | | | | | | | | | | | | | | |
| Azimi MW, 2019 | ANC | 18790 | | Wealth status  AOR, 95% CI; *p < 0.05, **p < 0.01, ***p < 0.001 | | | | | | | | | | | | | |
|  |  |  |  | Poor  Middle  Rich | | | | | | Ref.  1.32***  1.64*** | | | | | | | |
| Mumtaz S, 2019 | ANC, SBA | 19642 | | AOR, 95% CI; *p < 0.05, **p < 0.01, ***p < 0.001 | | | | | | | | | | | | | |
|  |  |  |  | Wealth status | | | | ANC | | | | SBA | | | | | |
|  |  |  |  | Poorest  Poorer  Middle  Richer  Richest | | | | Ref.  1.14 (0.86–1.58)  1.53 (1.12–2.08) ***  1.89 (1.42–2.53) ***  2.69 (1.81–3.98) *** | | | | Ref.  1.95 (1.67–2.29)  2.64 (2.05–3.41) ***  5.55 (4.37–7.05) ***  11.01(7.26–16.70) *** | | | | | |
| Akseer N, 2016 | ANC, SBA | 21290 | | Estimates with 95% CI | | | | 4+ ANC | | | | SBA | | | | | |
|  |  |  |  | SII | | | | 31.7 (26.2, 37.2) | | | | 65.4 (60.0, 70.8) | | | | | |
|  |  |  |  | Ratio (Q5:Q1) | | | | 5.57 (4.41, 8.31) | | | | 4.91 (4.18, 6.06) | | | | | |
| Higgins-Steele A, 2018 | ID | 894 | | Money for health services in three provinces, OR, 95% CI, P < 0.001 | | | | | | | | | | | | | |
|  |  |  |  | Badghi | | | | Bamyan | | | | Kandahar | | | | | |
|  |  |  |  | Ref. | | | | 0.110 (0.092–0.145) | | | | 0.750 (0.209–0.316) | | | | | |

AOR: Adjusted odds ratio; OR: Odds ratio; CI: Confidence interval; Ref: Reference; NS: Not significant

**Supplementary Table (S5): Quantitative results by education**

| **Country: India** | | | | | | | | | | | | | | | | | | | | | | | | | | |
| --- | --- | --- | --- | --- | --- | --- | --- | --- | --- | --- | --- | --- | --- | --- | --- | --- | --- | --- | --- | --- | --- | --- | --- | --- | --- | --- |
| Study | Indicator | Sample size | Quantitative finding | | | | | | | | | | | | | | | | | | | | | | | |
| Singh L, 2019 | ANC | 190898 | Years of schooling, OR, 95% CI, *p value significant at <0.001 | | | | | | | | | | | | | | | | | | | | | | | |
|  |  |  |  | | | | | | | | | | | | | | No & inadequate ANC vs adequate ANC | | | | | | | | | |
|  |  |  | No schooling  <5  5-7  8-9  10-11  >12 | | | | | | | | | | | | | | Ref.  1.23 (1.11, 1.36)*  1.33 (1.24, 1.46)*  1.45 (1.35, 1.56)*  1.50 (1.39, 1.62)*  1.55 (1.43, 1.67)* | | | | | | | | | |
| Pallikadavath S, 2004 | ANC | 11369 | OR, 95% CI; **p < 0.010, *p < 0.05 (Excluding home visits) | | | | | | | | | | | | | | | | | | | | | | | |
|  |  |  | Level | | | | | | | | Husband | | | | | | Wife | | | | | | | | | |
|  |  |  | Bihar | | | | | | | | | | | | | | | | | | | | | | | |
|  |  |  | Primary  Secondary  High | | | | | | | | 1.4  1.6**  2.5** | | | | | | 1.0  1.7**  2.9** | | | | | | | | | |
|  |  |  | Madhya Pradesh | | | | | | | | | | | | | | | | | | | | | | | |
|  |  |  | Primary  Secondary  High | | | | | | | | 1.2  1.2  1.7** | | | | | | 1.2  2.6  8.0** | | | | | | | | | |
|  |  |  | Rajasthan | | | | | | | | | | | | | | | | | | | | | | | |
|  |  |  | Primary  Secondary  High | | | | | | | | 1.5*  1.6*  1.9** | | | | | | 2.0*  2.2*  20.0** | | | | | | | | | |
|  |  |  | Uttar Pradesh | | | | | | | | | | | | | | | | | | | | | | | |
|  |  |  | Primary  Secondary  High | | | | | | | | 1.0  1.5**  1.6** | | | | | | 1.6**  2.3**  4.8** | | | | | | | | | |
| Sridharan S, 2017 | ANC | 5666 | OR, 95% CI; ***p < 0.01; **p < 0.05. | | | | | | | | | | | | | | | | | | | | | | | |
|  |  |  | 1.49*** (Women read or write)  1.30*** (Husband schooling) | | | | | | | | | | | | | | | | | | | | | | | |
| Zuhair M, 2017 | ANC | 36447 | OR, 95% CI; *p < 0.05 | | | | | | | | | | | | | | | | | | | | | | | |
|  |  |  | 1.10* (Education in years) | | | | | | | | | | | | | | | | | | | | | | | |
| Thakkar N, 2023 | ANC | 172702 | Education level, AOR, 95% CI, p-value significant at <0.05 | | | | | | | | | | | | | | | | | | | | | | | |
|  |  |  | Education level | | | | | | | | Inadequate ANC visits | | | | | | | | | p-value | | | | | | |
|  |  |  | No education  Primary  Secondary  Higher | | | | | | | | 1.76 (1.65–1.88)  1.21 (1.13–1.29)  1.07 (1.01–1.13)  Ref. | | | | | | | | | <0.001  <0.001  0.023  - | | | | | | |
| **Country: Nepal** | | | | | | | | | | | | | | | | | | | | | | | | | | |
| Chalise B, 2019 | ANC, SBA | 2086 | AOR; 95% CI; No education (Reference) | | | | | | | | | | | | | | | | | | | | | | | |
|  |  |  | Formal education | | | | | | | | ANC & SBA | | | | | | ANC, SBA & PNC | | | | | | | | | |
|  |  |  | Yes | | | | | | | | 1.45 (1.06,2.00) | | | | | | 1.46 (1.05,2.04) | | | | | | | | | |
| Khatiwada J, 2020 | SBA | 4400 | AOR; 95% CI; p value = 0.0000 | | | | | | | | | | | | | | | | | | | | | | | |
|  |  |  | SBA use | | | | | | | | | | | | | | | | | | | | | | | |
|  |  |  | No education  Primary  Secondary  Higher | | | | | | | | | | | | | | Ref.  1.41 (1.08 to 1.84)  2.09 (1.65 to 2.65)  2.65 (1.92 to 3.67) | | | | | | | | | |
| Dhakal S, 2011 | Skilled delivery service/ ID | 150 | Women’s education, AOR, 95% CI, *p < 0.05 | | | | | | | | | | | | | | | | | | | | | | | |
|  |  |  | Illiterate  Primary  Secondary | | | | | | | | | | | | | | Ref.  1.21 (0.35, 4.27)  5.61 (1.53, 20.54) | | | | | | | | | |
| Bhatta DN, 2015 | ANC, ID | 4803 | AOR, 95% CI, p < 0.001 | | | | | | | | | | | | | | | | | | | | | | | |
|  |  |  | Husband’s education | | | | | | | | ANC | | | | | | ID | | | | | | | | | |
|  |  |  | Illiterate & Primary  Secondary & Higher Secondary)  Graduate and above | | | | | | | | Ref.  2.66 (2.30, 3.55)  5.91 (4.02, 8.70) | | | | | | Ref.  5.93 (4.11, 8.55)  12.11 (7.64, 19.19) | | | | | | | | | |
| Khanal V, 2014 | PNC | 4079 | AOR, 95% CI; p < 0.001 | | | | | | | | | | | | | | | | | | | | | | | |
|  |  |  | Level | | | | | | | | Maternal | | | | | | Paternal | | | | | | | | | |
|  |  |  | No education  Primary  Secondary  Higher | | | | | | | | Ref.  1.469 (1.180, 1.828)  2.279 (1.754, 2.961)  4.623 (2.880, 7.421) | | | | | | Ref.  1.266 (0.967, 1.657)  1.489 (1.129, 1.965)  1.736 (1.099, 2.742) | | | | | | | | | |
| Joshi C, 2014 | ANC | 4079 | Adjusted OR, 95% CI; | | | | | | | | | | | | | | | | | | | | | | | |
|  |  |  | Level | | | | | | | | Women; p < 0.001 | | | | | | Husband; p = 0.010 | | | | | | | | | |
|  |  |  | No Education  Primary  Secondary  Tertiary | | | | | | | | Ref.  1.72 (1.38, 2.14)  2.50 (1.94, 3.22)  7.11 (3.28, 15.44) | | | | | | Ref.  1.55 (1.12, 2.13)  1.81 (1.31, 2.51)  1.60 (0.98, 2.61) | | | | | | | | | |
| Rahman MA 2021 | ID | 16429;  3962 (Nepal) | Education; AOR, 95% CI; *p<0.05, **p < 0.01, ***p < 0.001 | | | | | | | | | | | | | | | | | | | | | | | |
|  |  |  | Women’s Education | | | | | | | | | | | Husband’s Education | | | | | | | | | | | | |
|  |  |  | No-education  Primary  Secondary  Higher | | | | | Ref.  1.14(0.92,1.42)  1.55(1.23,1.94)***  2.56(1.80,3.64)*** | | | | | | No-education  Primary  Secondary  Higher | | | | | | | | | | Ref.  1.06(0.82,1.38)  1.12(0.87,1.44)  1.28(0.92,1.79) | | |
| Thapa B, 2023 | ID | 1932 | Women’s Education; AOR, 95% CI; *p<0.05, **p < 0.01, ***p < 0.001, AIC: Akaike Information Criterion (Best Fitting Model, Final Model) | | | | | | | | | | | | | | | | | | | | | | | |
|  |  |  | Formal education  No education | | | | | | | | | | | | 1.65(1.16–2.35) **  Ref. | | | | | | | | | | | |
|  |  |  | AIC | | | | | | | | | | | | 1484.94 | | | | | | | | | | | |
| **Country: Bangladesh** | | | | | | | | | | | | | | | | | | | | | | | | | | |
| Huda TM, 2019 | Health facility delivery/ ID | 28032 | Maternal education | | | | | | | | | | | | | | AOR, 95% CI | | | | | | | | | |
|  |  |  | No education  Incomplete primary  Complete primary  Secondary or higher | | | | | | | | | | | | | | Ref.  1.01 (0.89, 1.15)  1.03 (0.90, 1.18)  1.43 (1.27, 1.61) | | | | | | | | | |
| Dalal K, 2012 | ID | 4925 | AOR, 95% CI; ***p < 0.001, **p < 0.010, *p < 0.05 | | | | | | | | | | | | | | | | | | | | | | | |
|  |  |  | Level | | | | | | | | Maternal | | | | | | Paternal | | | | | | | | | |
|  |  |  | No education  Primary  Secondary  Higher | | | | | | | | 14.21 (5.09-37.98)***  6.33 (2.82–14.18)***  2.53 (1.29–4.96)**  Ref. | | | | | | 2.14 (0.91–5.06)  2.89 (1.35–6.21)**  1.63 (0.86–3.11)  Ref. | | | | | | | | | |
| Kamal SM, 2015 | SBA | 4809 | Women education  AOR, 95% CI; *p < 0.05, **p < 0.1, ***p < 0.001 | | | | | | | | | | | | | | | | | | | | | | | |
|  |  |  | No education  Primary  Secondary+ | | | | | | | | | | | | | | Ref.  1.02 (0.70-1.51)  1.96 (1.35-2.86)*** | | | | | | | | | |
| Pulok MH, 2016 | ANC, SBA, ID | 3730 (2004)  3365 (2007)  4648 (2011) | OR, 95% CI, *p < 0.1, **p < 0.05, ***p < 0.01 | | | | | | | | | | | | | | | | | | | | | | | |
|  |  |  | Education (in years | | | | 4+ANC | | | | | | | | | ID | | | | | | | SBA | | | |
|  |  |  | Mother | | | | 1.12*** | | | | | | | | | 1.12*** | | | | | | | 1.11*** | | | |
|  |  |  | Husband | | | | 1.05*** | | | | | | | | | 1.06*** | | | | | | | 1.06*** | | | |
| Chanda SK, 2020 | ANC | 4475 | Education, OR, 95% CI | | | | | | | | | | | | | | | | | | | | | | | |
|  |  |  | Maternal | | | | | | | | ANC contact | | | | | | 8+ ANC | | | | | | | | | |
|  |  |  | No Edu.  Primary  Secondary  Higher | | | | | | | | Ref.  1.35 (1.08, 1.70)  2.10 (1.63, 2.71)  3.29 (1.89, 5.73) | | | | | | Ref.  1.78 (0.87, 3.64)  2.03 (1.00, 4.13)  2.86 (1.30, 6.29) | | | | | | | | | |
|  |  |  | Husband | | | | | | | | ANC contact | | | | | | 8+ ANC | | | | | | | | | |
|  |  |  | Higher  Secondary  Primary  No Edu. | | | | | | | | Ref.  1.01 (0.83, 1.24)  1.49 (1.17, 1.90)  1.95 (1.26, 3.01) | | | | | | Ref.  NS  NS  NS | | | | | | | | | |
| Yaya S, 2017 | ID | 7313 | OR, 95% CI; (Conditional logistic regression); ***p < 0.05 | | | | | | | | | | | | | | | | | | | | | | | |
|  |  |  | Level | | | | | | | | Maternal | | | | | | Husband | | | | | | | | | |
|  |  |  | Nil  Primary  High | | | | | | | | Ref.  1.256 (0.995, 1.531)  2.081(1.650, 2.624) *** | | | | | | Ref.  0.999 (0.824, 1.213)  1.709 (1.412, 2.069) *** | | | | | | | | | |
| Bhowmik J, 2019 | ANC, SBA | 17863 | OR, 95% CI; Mixed effect model | | | | | | | | | | | | | | | | | | | | | | | |
|  |  |  | ANC | | | | | | | | | | | | | | | | | | | | | | | |
|  |  |  | Edu. Level | | | | | | | | Maternal | | | | | | Husband | | | | | | | | | |
|  |  |  | No edu.  Primary  Secondary  Higher | | | | | | | | Ref.  1.30 (1.02,1.66)  1.98 (1.52,2.58)  3.45 (2.05,5.83) | | | | | | Ref.  1.04 (0.85,1.28)  1.74 (1.37,2.21)  2.81 (1.87,4.23) | | | | | | | | | |
|  |  |  | SBA | | | | | | | | | | | | | | | | | | | | | | | |
|  |  |  | No edu.  Primary  Secondary  Higher | | | | | | | | Ref.  1.33 (1.00, 1.77)  1.87 (1.40, 2.51)  3.33 (2.20, 5.04) | | | | | | Ref.  1.20 (0.96, 1.50)  1.48 (1.17, 1.89)  2.52 (1.82, 3.50) | | | | | | | | | |
| Rahman MA 2021 | Facility based delivery | 16,429 (4278 in Bangladesh) | Education; AOR, 95% CI; *p<0.05, **p < 0.01, ***p < 0.001 | | | | | | | | | | | | | | | | | | | | | | | |
|  |  |  | Women’s Education | | | | | | | | | | | | Husband’s education | | | | | | | | | | | |
|  |  |  | No-education  Primary  Secondary  Higher | | | Ref.  1.80(1.37, 2.36) ***  2.17(1.58, 2.99) ***  2.28(1.54, 3.37) *** | | | | | | | | | No-education  Primary  Secondary  Higher | | | | | | | Ref.  1.17(0.94, 1.45)  1.26(1.00, 1.60)  1.73(1.27, 2.35) ** | | | | |
| Methun MIH  2022 | ANC, ID | 4012 | Education; desired OR; 95% CI; P-value<0.05*, P-value<0.01**, P-value<0.001*** | | | | | | | | | | | | | | | | | | | | | | | |
|  |  |  | No education  Primary  Secondary  Higher | | | | | | | | Ref.  1.421 (0.854, 2.365)  2.737 (1.709, 4.384)***  5.571 (3.437, 9.029)*** | | | | | | | | | | | | | | | |
| Misu F, 2023 | PNC | Bangladesh (4440) | RCI: Relative Concentration Index; ACI: Absolute Concentration Index; SII: Slope Index of inequality; CI: Confidence interval, significant at p≤0.05 | | | | | | | | | | | | | | | | | | | | | | | |
|  |  |  | Women’s Education | | | | | | | | PNC Check of Women within 2 Days by Skilled Provider | | | | | | | | | | | | | | | |
|  |  |  | RCI [95% CI]  ACI [95% CI]  SII [95% CI] | | | | | | | | 0.404 [0.370–0.437], p-value = 0.00  0.403 [0.370–0.437], p-value = 0.00  0.624 [0.579–0.670], p-value = 0.00 | | | | | | | | | | | | | | | |
| Misu F, 2023 | ANC, SBA, ID | Bangladesh (4948) | Education, RCI: Relative Concentration Index; ACI: Absolute Concentration Index; SII: Slope Index of inequality | | | | | | | | | | | | | | | | | | | | | | | |
|  |  |  | Women’s education | | | | | | At least 4 ANC visits by skilled provider | | | | | | | Skilled Birth Attendance (SBA) | | | | | Facility-Based Delivery | | | | | |
|  |  |  | RCI  ACI  SII | | | | | | 0.299  0.298  0.476 | | | | | | | 0.380  0.379  0.591 | | | | | 0.373  0.373  0.582 | | | | | |
|  |  |  | Husband’s education | | | | | |  | | | | | | |  | | | | |  | | | | | |
|  |  |  | RCI  ACI  SII | | | | | | 0.310  0.309  0.466 | | | | | | | 0.375  0.373  0.554 | | | | | 0.368  0.368  0.546 | | | | | |
| Methun MIH, 2023 | ANC, ID | Bangladesh (5012) | Shapley’s decomposition method for the relative contribution of education to the inequality | | | | | | | | | | | | | | | | | | | | | | | |
|  |  |  |  | | | | | | | | Access to minimum required ANC visits (4+ ANC) | | | | | | | Access to institutional  delivery | | | | | | | | |
|  |  |  | Education | | | | | | | | 31.4% | | | | | | | 27.4% | | | | | | | | |
| Jannat Z, 2023 | ANC, PNC | ANC= 245  PNC=  133 | Education level, AOR, 95% CI, p-value of < 0.05 | | | | | | | | | | | | | | | | | | | | | | | |
|  |  |  |  | | | | | | | | ANC 4+ visits | | | | | | | PNC 4+ visits | | | | | | | | |
|  |  |  |  |  |  |  |  |  |  |  | AOR, 95% CI | | | | p value | | | AOR, 95% CI | | | | | | | | p value |
|  |  |  | No education  Primary incomplete  Primary complete  Secondary incomplete  Secondary or above | | | | | | | | 0.7(0.3–2)  0.3(0.2–0.7)  0.6(0.3–1.1)  0.5(0.3–0.9)  Ref. | | | | 0.534  0.003  0.098  0.009  - | | | 5.3(1.3–21.9)  1.5(0.6–4.0)  1.6(0.6–4.0)  2.3(1.3–4.1)  Ref. | | | | | | | | 0.020  0.414  0.327  0.005  - |
| **Country: Pakistan** | | | | | | | | | | | | | | | | | | | | | | | | | | |
| Jain AK, 2015 | ID | 4435 | AOR, 95% CI; *p < .05; **p < .01; ***p < 001. | | | | | | | | | | | | | | | | | | | | | | | |
|  |  |  | Edu. Level | | | | | | | | Net effect | | | | | | Individual effect | | | | | | | | | |
|  |  |  | No  Primary  Secondary or higher | | | | | | | | Ref.  1.50**  2.62*** | | | | | | Ref.  1.50**  2.61*** | | | | | | | | | |
| Ghaffar A 2015 | ANC | 2339 | Akaike Information Criterion; p value = *0.1, **0.05, ***0.01; | | | | | | | | | | | | | | | | | | | | | | | |
|  |  |  | Factor | | | | | | | | Estimate | | | | | | Exp(estimate) | | | | | | | | | |
|  |  |  | Literacy | | | | | | | | 1.049*** | | | | | | 2.85 | | | | | | | | | |
| Sahito A, 2018 | ANC | 7142 | OR, 95% CI; [Gilgit Baltistan (GB), Khyber Pakhtunkhwa (KPK)] | | | | | | | | | | | | | | | | | | | | | | | |
|  |  |  | Maternal Edu | | GB | | | | | | | KPK | | | | | Sindh | | | | | | | | Punjab | |
|  |  |  | No Edu.  Primary  Secondary  Higher | | Ref.  3.2 (1.0, 10.4)  5.3 (2.6, 10.8)  2.5 (1.2, 5.4) | | | | | | | NS  NS  NS  NS | | | | | Ref.  1.4 (0.9, 2.2)  2.1 (1.3, 3.2)  6.6 (3.2, 13.6) | | | | | | | | Ref.  1.4 (1.0, 2.1)  2.1 (1.5, 2.9)  4.2 (2.3, 7.9) | |
|  |  |  | Paternal Edu | | | | | | | | | | | | | | | | | | | | | | | |
|  |  |  | No Edu.  Primary  Secondary  Higher | | NS  NS  NS  NS | | | | | | | Ref.  1.0 (0.6, 1.9)  2.0 (1.4, 2.8)  1.9 (1.2, 3.1) | | | | | NS  NS  NS  NS | | | | | | | | NS  NS  NS  NS | |
| Budhwani H, 2015 | ANC/ Prenatal, SBA, ID, PNC | 7399 | OR, 95% CI; *p < 0.05, **p < 0.01, ***p < 0.001 | | | | | | | | | | | | | | | | | | | | | | | |
|  |  |  | Factor | | | | | | | | 4+ ANC/ prenatal | | | | | | ID | | | | SBA | | | | | PNC |
|  |  |  | Mothers Edu. | | | | | | | | 1.966*** | | | | | | 1.599*** | | | | 1.647*** | | | | | 1.228* |
| Rahman MA 2021 | Facility based delivery | 16,429 (8189 in Pakistan) | Education; AOR, 95% CI; *p<0.05, **p < 0.01, ***p < 0.001 | | | | | | | | | | | | | | | | | | | | | | | |
|  |  |  | Women’s Education | | | | | | | | | | | | | Husband’s Education | | | | | | | | | | |
|  |  |  | No edu  Primary  Secondary  Higher | Ref.  1.16(0.98, 1.37)  1.44(1.21, 1.70) ***  2.83(2.15, 3.70) *** | | | | | | | | | | | | No edu  Primary  Secondary  Higher | | | Ref.  1.14(0.96–1.35)  1.04(0.90, 1.20)  1.19(0.99, 1.43) | | | | | | | |
| Misu F, 2023 | PNC | Pakistan (3780) | RCI: Relative Concentration Index; ACI: Absolute Concentration Index; SII: Slope Index of inequality; CI: Confidence interval, significant at p≤0.05 | | | | | | | | | | | | | | | | | | | | | | | |
|  |  |  | Women’s education | | | | | | | | PNC Check of Women within 2 Days by Skilled Provider | | | | | | | | | | | | | | | |
|  |  |  | RCI [95% CI]  ACI [95% CI]  SII [95% CI] | | | | | | | | 0.389 [0.333–0.444], p-value = 0.00  0.388 [0.333–0.444], p-value = 0.00  0.676 [0.605–0.747], p-value = 0.00 | | | | | | | | | | | | | | | |
| Misu F, 2023 | ANC, SBA, ID | Pakistan (5122) | Education, RCI: Relative Concentration Index; ACI: Absolute Concentration Index; SII: Slope Index of inequality | | | | | | | | | | | | | | | | | | | | | | | |
|  |  |  | Women’s education | | At least four ANC visits by skilled provider | | | | | | | | Skilled Birth Attendance | | | | | | | Facility-Based Delivery | | | | | | |
|  |  |  | RCI  ACI  SII | | 0.470  0.470  0.757 | | | | | | | | 0.394  0.308  0.611 | | | | | | | 0.392  0.327  0.627 | | | | | | |
|  |  |  | Husband’s education | | | | | | | | | | | | | | | | | | | | | | | |
|  |  |  | RCI  ACI  SII | | 0.380  0.379  0.572 | | | | | | | | 0.332  0.260  0.424 | | | | | | | 0.335  0.280  0.451 | | | | | | |
| **Country: Afghanistan** | | | | | | | | | | | | | | | | | | | | | | | | | | |
| Azimi MW, 2019 | ANC | 18790 | AOR, 95% CI; *p < 0.05, **p < 0.01, ***p < 0.001 | | | | | | | | | | | | | | | | | | | | | | | |
|  |  |  | Level | | | | | | | | Maternal | | | | | | Husband | | | | | | | | | |
|  |  |  | No education  Education | | | | | | | | Ref.  1.45*** | | | | | | Ref.  1.42*** | | | | | | | | | |
| Mumtaz S, 2019 | ANC, SBA | 19642 | AOR, 95% CI; *p < 0.05, **p < 0.01, ***p < 0.001 | | | | | | | | | | | | | | | | | | | | | | | |
|  |  |  | Level | | | | | | | | ANC | | | | | | SBA | | | | | | | | | |
|  |  |  | No education  Primary  Secondary  Higher | | | | | | | | Ref.  1.78 (1.37–2.31) ***  2.26 (1.55–3.29) ***  3.70 (2.14–6.41) *** | | | | | | Ref.  2.32 (1.80–2.98) ***  2.79 (2.18–3.57) ***  15.52 (6.39–27.37) *** | | | | | | | | | |
| Yeo S 2022 | ANC | 11056 | AOR, 95% CI; *p < 0.05, **p < 0.01, ***p < 0.001 | | | | | | | | | | | | | | | | | | | | | | | |
|  |  |  | No Education  Primary  Secondary  Higher  Literacy | | | | | | | | Ref.  1.67*(1.02, 2.72)  2.43**(1.25, 4.70)  3.03** (1.30, 7.07)  0.66** (0.40, 1.09) | | | | | | | | | | | | | | | |
| Stanikzai M.H., 2023 | ANC | 6227 | Literacy level in receiving 5–8 ANC services during a single ANC visit;  AOR; 95% CI | | | | | | | | | | | | | | | | | | | | | | | |
|  |  |  | Reads and writes | | | | | | | Women; p = < 0.001 | | | | | | | | | | | Husband; p=0.04 | | | | | |
|  |  |  | No  Yes | | | | | | | Ref.  1.33 (1.15–1.54) | | | | | | | | | | | Ref.  1.14 (1.00-1.28) | | | | | |

AOR: Adjusted odds ratio; OR: Odds ratio; CI: Confidence interval; Ref: Reference; NS: Not significant

**Supplementary Table (S6): Quantitative results by occupation**

| **Country: India** | | | | | | | | | | | | |
| --- | --- | --- | --- | --- | --- | --- | --- | --- | --- | --- | --- | --- |
| Study | Indicator | Sample size | Quantitative findings | | | | | | | | | |
| Sridharan, S, 2017 | ANC | 5666 | OR, 95% CI, ***p < 0.01; **p < 0.05. | | | | | | | | | |
|  |  |  | 1.39*** (Working) | | | | | | | | | |
| Pallikadavath S, 2004 | ANC | 11369 | OR (95% CI or significance), **p < 0.010; *p < 0.05 | | | | | | | | | |
|  |  |  | States | | Working status (Yes) | | | | | | | |
|  |  |  |  |  | Including home visit | | Excluding home visit | | | | | |
|  |  |  | Bihar  Madhya Pradesh  Rajasthan  Uttar Pradesh | | 0.9  1.1  1.1  1.2* | | 0.8  0.9  1.1  1.2* | | | | | |
| Khatiwada J, 2020 | SBA | 4400 | AOR, 95% CI; p value = 0.000 | | | | | | | | | |
|  |  |  | SBA use | | | | | | | | | |
|  |  |  | Not working  Agriculture  Other | | | | Ref.  0.66 (0.05, 0.81)  1.06 (0.80, 1.40) | | | | | |
| Joshi C, 2014 | ANC | 4079 | Husband’s occupation  Adjusted OR; 95% CI; p = 0.002 | | | | | | | | | |
|  |  |  | Agriculture  Professional/ Technical/ Manager  Clerical  Services  Skilled manual  Unskilled manual  Other | | | | Ref.  2.13 (1.27, 3.58)  1.44 (0.96, 2.14)  1.76 (1.29, 2.39)  1.36 (1.02, 1.82)  1.47 (1.05, 2.05)  0.83 (0.42, 1.63) | | | | | |
| **Country: Bangladesh** | | | | | | | | | | | | |
| Bhowmik J, 2019 | ANC, SBA | 17863 | OR, 95% CI; Mixed effect model | | | | | | | | | |
|  |  |  | Working status | ANC | | | SBA | | | | | |
|  |  |  | No  Yes | Ref.  0.92 (0.76, 1.11) | | | Ref.  0.76 (0.63, 0.92) | | | | | |
| Dalal K, 2012 | ID | 4925 | AOR, 95% CI; ***p < 0.001, **p < 0.010, *p < 0.05 | | | | | | | | | |
|  |  |  | Not working  Working | | | | 1.87 (0.84,4.17)  Ref. | | | | | |
| Pulok MH, 2016 | ANC, SBA, ID | 3730 (2004);  3365 (2007);  4648 (2011) | OR, 95% CI; *p < 0.1; **p < 0.05; ***p < 0.01 | | | | | | | | | |
|  |  |  | Employment | ANC | | | ID | | | | SBA | |
|  |  |  | No  Yes | Ref.  0.86 (0.72, 1.03) | | | Ref.  0.77* (0.63, 0.95) | | | | Ref.  0.82* (0.67, 0.99) | |
| Rahman MA 2021 | Facility based delivery | 16429  (4278  in Bangladesh) | AOR, 95% CI; *p < 0.05; **p < 0.01; ***p < 0.01 | | | | | | | | | |
|  |  |  | Women’s occupation | | | Husband’s Occupation | | | | | | |
|  |  |  | Not-working  Working | Ref.  0.77(0.65, 0.923) * | | Agriculture  Professional/ Service  Others | | | | | Ref.  1.30(1.04, 1.61)*  0.93(0.76, 1.23) | |
| Methun MIH 2022 | ANC, ID | 4012 | Working Status; OR, 95% CI; *p < 0.05; **p < 0.01; ***p < 0.01 | | | | | | | | | |
|  |  |  | Working  Not-working | | | Ref.  1.124 | | | | | | |
| Misu F, 2023 | ANC, SBA, ID | Bangladesh (4948) | Women’s employment status; Rate Ratio; Rate Difference | | | | | | | | | |
|  |  |  | Employment status | At least four ANC visits by skilled provider | | Skilled Birth Attendance (SBA) | | | | | | Facility-based delivery |
|  |  |  | Rate ratio (ref:  not currently  employed) | 1.058 | | 1.314 | | | | | | 1.348 |
|  |  |  | Rate difference  (ref: not currently  employed) | 0.026 | | 0.139 | | | | | | 0.142 |
| **Country: Nepal** | | | | | | | | | | | | |
| Dhakal S, 2007 | PNC | 150 | AOR, 95% CI [Kathmandu] | | | | | | | | | |
|  |  |  | Women’s occupation | | | | | | | | | |
|  |  |  | Farmer  Housewife  Other | | | | Ref.  6.28 (2.00, 19.69)  3.06 (0.27, 34.64) | | | | | |
|  |  |  | Husband’s occupation | | | | | | | | | |
|  |  |  | Farmer  Formal sector + worked abroad  Other | | | | Ref.  0.83 (0.27, 2.53)  0.15 (0.03, 0.85) | | | | | |
| Khanal V, 2014 | PNC | 4079 | Adjusted OR, 95% CI | | | | | | | | | |
|  |  |  | Maternal occupation; p = 0.002 | | | | | | | | | |
|  |  |  | Not working  Agriculture  Working, paid | | | | Ref.  0.623 (0.481, 0.807)  0.782 (0.578, 1.052) | | | | | |
|  |  |  | Paternal occupation; p <0.001 | | | | | | | | | |
|  |  |  | Not working  Professional/ technical  Manual | | | | Ref.  1.718 (1.354, 2.179)  1.398 (1.102, 1.772) | | | | | |
| Rahman MA 2021 | Facility delivery | 16429 (3962 in Nepal) | Occupation; AOR, 95% CI; ***p < 0.001, **p < 0.010, *p < 0.05 | | | | | | | | | |
|  |  |  | Mother’s Occupation | | | Husband’s Occupation | | | | | | |
|  |  |  | Not Working  Working | Ref.  0.85(0.72–1.01) | | Agricultural  Professional/ Services  Others | | | | | Ref.  1.26(1.01, 1.58)*  1.09(0.88, 1.34) | |
| **Country: Pakistan** | | | | | | | | | | | | |
| Sahito A, 2018 | ANC | 7142 | Employment (Husband) | | | OR, 95% CI (Sindh Province) | | | | | | |
|  |  |  | Not employed/unskilled  Skilled/non-manual  Professional/technical | | | Ref.  1.4 (1.0, 1.9)  1.2 (0.7, 2.0) | | | | | | |
|  |  |  | Women’s Occupation | | | | | | Husband’s Occupation | | | |
| Rahman MA 2021 | Facility based delivery | 16,429 (8189 in Pakistan) | AOR, 95% CI; *p < 0.05; **p < 0.01; ***p < 0.01 | | | | | | | | | |
|  |  |  | Husbands Occupation | | | | | | | | | |
|  |  |  | Agriculture  Professional/ Service  Others | | | | | | Ref.  0.86(0.57–1.29)  0.98(0.74–1.30) | | | |
| Misu F, 2023 | ANC, SBA, ID | Pakistan (5122) | Women’s employment status; Rate Ratio; Rate Difference | | | | | | | | | |
|  |  |  | Employment Status | At least four  ANC visits by  skilled provider | | Skilled Birth Attendance (SBA) | | | | Facility-based  Delivery | | |
|  |  |  | Rate ratio (ref: not currently  employed) | 1.187 | | 1.084 | | | | 1.076 | | |
|  |  |  | Rate difference  (ref: not currently  employed) | 0.084 | | 0.057 | | | | 0.050 | | |
| **Country: Afghanistan** | | | | | | | | | | | | |
| Mumtaz S, 2019 | ANC, SBA | 19642 | AOR, 95% CI; *p < 0.05, **p < 0.01, ***p < 0.001 | | | | | | | | | |
|  |  |  | Working status | ANC | | | | SBA | | | | |
|  |  |  | Not working  Working | Ref.  0.84 (0.69–1.04) *** | | | | Ref.  1.06 (0.87–1.36) *** | | | | |

AOR: Adjusted odds ratio; OR: Odds ratio; CI: Confidence interval; Ref: Reference; NS: Not significant

**Supplementary Table (S7): Quantitative results by women autonomy**

| **Country: India** | | | | | | | | | | | | | |
| --- | --- | --- | --- | --- | --- | --- | --- | --- | --- | --- | --- | --- | --- |
| Study | Indicator | Sample size | Quantitative findings | | | | | | | | | | |
| Pallikadavath S, 2004 | ANC | 11369 | OR (95% CI or significance), **p < 0.010; *p < 0.05 [Excluding home visits] | | | | | | | | | | |
|  |  |  | States | | | | | High autonomy | | | | | |
|  |  |  |  |  |  |  |  | Including home visit | | | | Excluding home visit | |
|  |  |  | Bihar  Madhya Pradesh  Rajasthan  Uttar Pradesh | | | | | 1.2  1.0  1.4  1.1 | | | | 1.3  1.4*  1.4  1.3* | |
| Mistry R, 2009 | PNC, SBA, ID | 11648 | *p < 0.05, **p < 0.01, ***p < 0.001 | | | | | | | | | | |
|  |  |  | Financial autonomy | | | | | | | | | | |
|  |  |  | North | | | | | | 1.14 (SBA)  1.22* (ID)  1.18 (PNC) | | | | |
|  |  |  | East | | | | | | 1.16 (SBA)  1.26* (ID)  1.09 (PNC) | | | | |
|  |  |  | South | | | | | | 1.86*** (SBA)  1.64*** (ID)  1.29* (PNC) | | | | |
| **Country: Nepal** | | | | | | | | | | | | | |
| Kc S, 2016 | ANC, SBA | 4148 | Women’s autonomy and skilled attendance during antenatal and delivery care, AOR, 95 % CI, P < 0.05 | | | | | | | | | | |
|  |  |  | Autonomy | | | | | ANC | | | | SBA | |
|  |  |  | Health care  No  Yes | | | | | Ref.  1.17 (1.02–1.35) | | | | Ref.  1.33(1.14–1.56) | |
|  |  |  | Large household purchase  No  Yes | | | | | Ref.  1.25 (1.09, 1.44) | | | | Ref.  1.25(1.07, 1.45) | |
|  |  |  | Spending money earned by husband  No  Yes | | | | | Ref.  1.30 (1.13, 1.49) | | | | Ref.  1.31(1.12, 1.54) | |
|  |  |  | Overall autonomy | | | | |  | | | |  | |
|  |  |  | Low | | | | | Ref. | | | | Ref. | |
|  |  |  | Medium | | | | | 1.20 (1.02, 1.40) | | | | 1.02 (0.86, 1.22) | |
|  |  |  | High | | | | | 1.33 (1.10, 1.59) | | | | 1.38 (1.12, 1.70) | |
| Rahman MA 2021 | Facility | 16,429 (3962 in Nepal) | Decision Making Power on Respondent’s Health Care; aOR, 95% CI, *p < 0.1, **p < 0.05, ***p < 0.01 | | | | | | | | | | |
|  |  |  | Someone Else  Self  Both (Wife & Husband)  Husband Alone | | | | | | | | | Ref.  1.10(0.86–1.40)  1.04(0.83–1.30)  1.08(0.86–1.34) | |
| **Country: Bangladesh** | | | | | | | | | | | | | |
| Pulok MH, 2016 | ANC, SBA, ID | 3730 (2004)  3365 (2007)  4648 (2011) | OR, 95% CI, *p < 0.1, **p < 0.05, ***p < 0.01 | | | | | | | | | | |
|  |  |  | Healthcare decision making | | | 4+ ANC | | | | ID | | | SBA |
|  |  |  | No  Low  Medium  High | | | Ref.  1.21*  1.36**  1.53*** | | | | Ref.  1.04  1.00  1.05 | | | Ref.  1.09  1.06  1.12 |
| Dalal K, 2012 | ID | 4925 | Who decides how to spend money | | | | AOR, 95% CI; ***p < 0.001, **p < 0.010, *p < 0.05 | | | | | | |
|  |  |  | Respondent alone  Shared  Others | | | | 0.76 (0.36, 1.57)  1.01 (0.50, 2.03)  Ref. | | | | | | |
| Bhowmik J, 2019 | ANC, SBA | 17863 | OR, 95% CI; Mixed effect model | | | | | | | | | | |
|  |  |  | Participation in household decision | | | | ANC | | | | | SBA | |
|  |  |  | No  Yes | | | | Ref.  0.81 (0.68, 0.96) | | | | | Ref.  0.78 (0.66, 0.93) | |
| Rahman MA 2021 | ID | 16,429 (4278 in Bangladesh) | Decision Making Power on Respondent’s Health Care; aOR, 95% CI, *p < 0.1, **p < 0.05, ***p < 0.01 | | | | | | | | | | |
|  |  |  | Someone Else  Self  Both (Wife & Husband)  Husband Alone | | | | | | | | | Ref.  0.95(0.68–1.33)  1.09(0.83–1.45)  1.04(0.78–1.39) | |
| Methun MIH 2022 | ANC, ID | 4012 | Health Care Decision; OR, 95% CI; *p < 0.05; **p < 0.01; ***p < 0.001 | | | | | | | | | | |
|  |  |  | Others (ref)  Self  Partner/Husband | | | | | | | | Ref.  1.18 (0.88-1.58)  1.23 (1.04-1.45)** | | |
| Misu F, 2023 | PNC | Bangladesh (4440) | Relative and Absolute Inequality Measure of PNC Services  RR: Rate ratio; RD: Rate difference, ref: reference category | | | | | | | | | | |
|  |  |  | Women’s autonomy | PNC Check of Women within 2 Days by Skilled Provider | | | | | | | | | |
|  |  |  | RR (ref: Yes)  RD (ref: Yes) | 1.005  0.002 | | | | | | | | | |
| **Country: Pakistan** | | | | | | | | | | | | | |
| Rahman MA 2021 | Facility | 16,429 (8189 in Pakistan) | Decision Making Power on Respondent’s Health Care; aOR, 95% CI, *p < 0.1, **p < 0.05, ***p < 0.01 | | | | | | | | | | |
|  |  |  | Someone Else  Self  Both (Wife & Husband)  Husband Alone | | | | | | | | | Ref.  0.95(0.74–1.23)  1.02(0.85–1.22)  0.88(0.74–1.04) | |
| Misu F, 2023 | PNC | Pakistan (3812) | Relative and Absolute Inequality Measure of PNC Services  RR: Rate ratio; RD: Rate difference, ref: reference category | | | | | | | | | | |
|  |  |  | Women’s autonomy | | PNC Check of Women within 2 Days by Skilled Provider | | | | | | | | |
|  |  |  | RR (ref: Yes)  RD (ref: Yes) | | 1.379  0.165 | | | | | | | | |
| **Country: Afghanistan** | | | | | | | | | | | | | |
| Mumtaz S, 2019 | ANC, SBA | 19642 | AOR, 95% CI; *p<0.05, **p < 0.01, ***p < 0.001 | | | | | | | | | | |
|  |  |  | Decision making autonomy on husband’s earning | | | | 4+ ANC | | | | | SBA | |
|  |  |  | No | | | | Ref. | | | | | Ref. | |
|  |  |  | Yes | | | | 1.53 (1.23–1.91) *** | | | | | 1.31 (1.12–1.52) *** | |
| Yeo S 2022 | ANC | 11056 | AOR, 95% CI; *p < 0.05, **p < 0.01, ***p < 0.001 | | | | | | | | | | |
|  |  |  | Decision-making (Alone or jointly) | | | | | | | | 1.16*** (1.08, 1.24) | | |

AOR: Adjusted odds ratio; OR: Odds ratio; CI: Confidence interval; Ref: Reference

**Supplementary Table (S8): Qualitative results by economy**

| Study | Study name | State | Result |
| --- | --- | --- | --- |
| India | | | |
| Vildler M, 2016 | Utilization of maternal health care services and their determinants in Karnataka State, India | Karnataka | Financial constraint; not for emergency but for routine care |
| Mahapatro M, 2015 | Equity in utilization of health care services: Perspective of pregnant women in southern Odisha, India | Odisha | Hospital staffs biased against rural poor women |
| Nepal | | | |
| Milne L, 2015 | Staff perspectives of barriers to women accessing birthing services in Nepal: a qualitative study | Kathmandu | Financial constraint for birthing facility |
| Lama S, 2014 | Barriers in Utilization of Maternal Health Care Services: Perceptions of Rural Women in Eastern Nepal | Jhapa, Nepal | Economic barrier  Transportation cost |
| Bangladesh | | | |
| Afsana K, 2004 | The tremendous cost of seeking hospital obstetric care in Bangladesh | Apurbabari | Tremendous unofficial costs;  Corruption |
| Pitchforth E, 2006 | Getting women to hospital is not enough: a qualitative study of access to emergency obstetric care in Bangladesh | Dhaka | Financial barrier in accessing EmOC;  Unofficial payments |
| Pakistan | | | |
| Nisar YB  2016 | Qualitative exploration of facilitating factors and barriers to use of antenatal care services by pregnant women in urban and rural settings in Pakistan | Swabi | Financial limitation  Travel cost  Consultation fee in private facility was expensive |
| Mumtaz Z, 2014 | Improving maternal health in Pakistan: toward a deeper understanding of the social determinants of poor women's access to maternal health services | Chakwal, North Punjab | Poverty was a reason for not accessing SBA and ID  Unable to pay for under-the-table payment  Transport cost |
| Memon Z, 2015 | Residual Barriers for Utilization of Maternal and Child Health Services: Community Perceptions from Rural Pakistan | Sindh | Unaffordability for transportation cost, medicine was the reason for not utilizing ANC, PNC, ID |
| Ansari MS, 2015 | Access to comprehensive emergency obstetric and newborn care facilities in three rural districts of Sindh province, Pakistan | Sindh | Poverty hampered access to C-EmONC,  Transportation cost,  Emegency treatment cost was unaffordable,  Less preference to poor people by hospital staff |
| Afghanistan | | | |
| Rahmani Z, 2013 | Antenatal and obstetric care in Afghanistan--a qualitative study among health care receivers and health care providers | Kabul & Ghazni | Poor economic condition was an obstacle for not utilizing MHC |
| Higgins-Steele A, 2018 | Barriers associated with care-seeking for institutional delivery among rural women in three provinces in Afghanistan | Badghis, Bamyan, and Kandahar | Main barrier in utilizing ID was cost |

**Supplementary Table (S9): Qualitative results by education**

| Study | Study name | State | Result |
| --- | --- | --- | --- |
| India | | | |
| Mahapatro M, 2015 | Equity in utilization of health care services: Perspective of pregnant women in southern Odisha, India | Odisha | Hospital staff are biased against the rural illiterate women |
| Nepal | | | |
| Lama S, 2014 | Barriers in Utilization of Maternal Health Care Services: Perceptions of Rural Women in Eastern Nepal | Jhapa, Nepal | Illiteracy and lack of awareness about the service |
| Milne L, 2015 | Staff perspectives of barriers to women accessing birthing services in Nepal: a qualitative study | Kathmandu | Lack of education  Unaware of the service and its importance |
| Pakistan | | | |
| Nisar YB | Qualitative exploration of facilitating factors and barriers to use of antenatal care services by pregnant women in urban and rural settings in Pakistan | Swabi | Lack of education was stated as a reason for not utilizing ANC |
| Memon Z, 2015 | Residual Barriers for Utilization of Maternal and Child Health Services: Community Perceptions from Rural Pakistan | Sindh | Lack of awareness about the importance of ANC, PNC and ID |
| Ansari MS, 2015 | Access to comprehensive emergency obstetric and newborn care facilities in three rural districts of Sindh province, Pakistan | Sindh | Less preference to illiterate people by hospital staff |

**Supplementary Table (S10): Qualitative results by women’s autonomy**

| Study | Study name | State | Result |
| --- | --- | --- | --- |
| India | | | |
| Vildler M, 2016 | Utilization of maternal health care services and their determinants in Karnataka State, India | Karnataka | Mothers-in-law and husbands hold the decision-making power, and they often resist facility-based care. |
| Mahapatro M, 2015 | Equity in utilization of health care services: Perspective of pregnant women in southern Odisha, India | Odisha | Women did not hold the decision-making autonomy; it was mostly done by the elders |
| Nepal | | | |
| Lama S, 2014 | Barriers in Utilization of Maternal Health Care Services: Perceptions of Rural Women in Eastern Nepal | Jhapa, Nepal | Husband restricts to go to the hospital |
| Milne L, 2015 | Staff perspectives of barriers to women accessing birthing services in Nepal: a qualitative study | Kathmandu Valley | Women’s illiteracy, unawareness and financial constraints are the barriers to avail proper MHC. |
| Pakistan | | | |
| Nisar YB, 2016 | Qualitative exploration of facilitating factors and barriers to use of antenatal care services by pregnant women in urban and rural settings in Pakistan | Swabi | No autonomy on healthcare decision; restricted by mother-in-law and husbands from going for ANC check ups |
| Afghanistan | | | |
| Rahmani Z, 2013 | Antenatal and obstetric care in Afghanistan--a qualitative study among health care receivers and health care providers | Kabul & Ghazni | Decision maker in the family mother-in-law or husband, not the mother;  They are not allowed to utilize MHC unless it is very serious |
